# Effect of dietary anthocyanins on the risk factors related to metabolic syndrome: A systematic review and meta-analysis

**DOI:** 10.1371/journal.pone.0315504

**Published:** 2025-02-10

**Authors:** Junyin Pan, Jingwen Liang, Zhantu Xue, Xin Meng, Liwei Jia

**Affiliations:** 1 School of Pharmacy of Heilongjiang University of Chinese Medicine, Harbin, Heilongjiang, China; 2 Foshan Clinical Medical School of Guangzhou University of Chinese Medicine, Guangzhou, Guangdong, China; Zanjan University of Medical Sciences, ISLAMIC REPUBLIC OF IRAN

## Abstract

**Objective:**

This meta-analysis aims to systematically investigate whether dietary anthocyanin supplementation can reduce metabolic syndrome (MetS)-related risk factors: abdominal obesity, dyslipidemia (low high-density lipoprotein cholesterol (HDL-C) and hypertriglyceridemia), hypertension, and hyperglycemia by conducting a meta-analysis of randomized controlled trials (RCTs).

**Methods:**

A systematic search of 5 electronic databases (PubMed, Web of Science, Scopus, Cochrane Library, and Embase) was conducted from inception until April 25, 2024. A total of 1213 studies were identified, of which randomized controlled trials involving subjects with MetS-related factors, comparing dietary anthocyanin supplementation with placebo, and reporting results on anthropometric, physiological, and metabolic markers relevant to this study were selected. Depending on the heterogeneity of the included studies, a fixed-effect model was applied for low heterogeneity (I^2^ < 50%), whereas a random-effects model was employed when substantial heterogeneity was present (I^2^ ≥ 50%). The weighted mean difference (WMD) and 95% confidence intervals (CI) were calculated.

**Results:**

This meta-analysis included 29 randomized controlled trials with 2006 participants. The results showed that dietary anthocyanins significantly improved various lipid and glycemic markers:

HDL-C: increased by 0.05 mmol/L (95% CI: 0.01 to 0.10, p = 0.026), LDL-C: decreased by 0.18 mmol/L (95% CI: -0.28 to -0.08, p = 0.000), Triglycerides (TGs): reduced by 0.11 mmol/L (95% CI: -0.20 to -0.02, p = 0.021), Total cholesterol (TC): lowered by 0.34 mmol/L (95% CI: -0.49 to -0.18, p = 0.000), Fasting blood glucose (FBG): reduced by 0.29 mmol/L (95% CI: -0.46 to -0.12, p = 0.001), Glycated hemoglobin (HbA1c): decreased by 0.43% (95% CI: -0.74 to -0.13, p = 0.005). Weight: (WMD: -0.12 kg, 95% CI: -0.45 to 0.21, p = 0.473), Body mass index (BMI): (WMD: -0.12 kg/m^2^, 95% CI: -0.26 to 0.03, p = 0.12), Overall WC: (WMD: 0.18 cm, 95% CI: -0.51 to 0.87, p = 0.613), Systolic blood pressure (SBP): (WMD: -0.12 mmHg, 95% CI: -1.06 to 0.82, p = 0.801), Diastolic blood pressure (DBP): (WMD: 0.61 mmHg, 95% CI: -0.03 to 1.25, p = 0.061), Insulin levels: (WMD: -0.02 mU/L, 95% CI: -0.44 to 0.40, p = 0.932), HOMA-IR: (WMD: -0.11, 95% CI: -0.51 to 0.28, p = 0.573). Additionally, a 100 mg/day dosage of anthocyanins significantly reduced: Waist circumference (WC): by 0.55 cm (95% CI: -1.09 to -0.01, p = 0.047). Subgroup analyses based on intervention duration, anthocyanin dosage, health status, formulation, dosage frequency, physical activity levels, and baseline levels of corresponding markers revealed varying significances, particularly in relation to blood pressure.

**Conclusion:**

Dietary anthocyanins effectively improve low HDL cholesterol, hypertriglyceridemia, and hyperglycemia, making them a promising adjunct for managing MetS. However, it is important to note that dietary anthocyanin interventions may raise systolic blood pressure (SBP) and diastolic blood pressure (DBP) depending on intervention dose, duration, participant health status, and formulation. Clinicians should fully consider these effects when recommending anthocyanin supplementation. Further long-term, well-designed, large-scale clinical trials are needed to draw definitive conclusions.

## Introduction

Metabolic syndrome (MetS) represents a complex clinical condition encompassing a constellation of interrelated factors, namely abdominal obesity, insulin resistance, hypertension, and dyslipidemia [1, 2]. Globally, the prevalence of metabolic syndrome (MetS) has risen significantly, affecting an estimated one billion people, with rates ranging from 12.5% to 31.4% depending on the diagnostic criteria. In the United States, approximately one-third of adults have MetS, while in China, prevalence increased to around 15.5% by 2017, posing a major public health challenge due to its association with high morbidity and mortality [3, 4]. This multifactorial syndrome significantly heightens the risk of developing cardiovascular diseases and type 2 diabetes [5–7]. Moreover, MetS is intricately linked to an increased incidence of several malignancies, including but not limited to, cancers of the colon, liver, and pancreas [8]. Numerous studies have demonstrated that the impact of each component of MetS on the overall syndrome is cumulative, and the risk of cardiovascular diseases and type 2 diabetes escalates concomitantly with an increase in the number of MetS components [8]. Reports indicate that individuals with MetS presenting four or more components are at an approximately 35-fold higher risk of developing Type 2 Diabetes Mellitus (T2DM) compared to the general population [9]. MetS, as an emerging non-communicable disease, has escalated into a global concern. The prevalence of MetS and its associated cardiometabolic components is widespread internationally. Managing the aberrant metabolic profile in patients with MetS is of paramount importance; however, the pharmacological options capable of effectively ameliorating the comprehensive metabolic dysfunctions associated with MetS are notably limited. While statins are widely regarded as pivotal in treating MetS, their use is frequently accompanied by adverse effects such as hepatotoxicity, nephrotoxicity, and rhabdomyolysis [10].

Although healthcare organizations worldwide adopt varying definitions and emphasize different aspects of MetS [3], the majority of these criteria for diagnosing MetS converge on the presence of at least three out of five core components: obesity, hyperglycemia, high blood pressure, reduced levels of serum High-Density Lipoprotein Cholesterol (HDL-C), and increased serum triglyceride (TG) levels [11]. However, by ameliorating these factors, not only can MetS be prevented, but the reduction of one or two components of MetS may also potentially diminish the risk of Cardiovascular Diseases (CVD) and T2DM [8]. In reality, this objective can be achieved through the adoption of more scientific dietary habits, including the integration of suitable dietary supplements. Notably, a wide range of natural products exhibit extensive pharmacological activities. In recent years, many researchers have evaluated the effectiveness of natural products and herbs containing active compounds on various chronic diseases such as diabetes, high blood pressure, metabolic syndrome, and cardiovascular diseases, and positive results have been found in some of these studies [12–14]. These substances act through various mechanisms on the pathophysiological signaling pathways of MetS, thereby ameliorating the components associated with MetS [15].

Anthocyanins, water-soluble pigments within the flavonoid family, are ubiquitously distributed across nature and human diets, with over 90% based on six primary anthocyanins: pelargonidin (Pg), cyanidin (Cn), delphinidin (Dp), peonidin (Pn), petunidin (Pt), and malvidin (Mv). These compounds impart red, blue, and purple hues to vegetables, fruits, and beverages, such as strawberries, cherries, cranberries, blueberries, red cabbage, eggplants, and red wine [16, 17]. Recent studies have illuminated the roles of anthocyanins in exhibiting antioxidant, anti-inflammatory, anti-atherosclerotic, cancer-preventive, and diabetes-preventive properties [18, 19]. These capabilities position anthocyanins as preventative and therapeutic agents against a spectrum of chronic conditions [20, 21], including MetS [22], CVD [23], ocular disorders [24], and osteoporosis [25]. As highlighted in recent research, anthocyanins have emerged as potential pharmacological agents in the treatment of MetS [26].

Previous research has documented that anthocyanin supplementation can improve inflammation levels, glycemic parameters, lipid profiles, and cardiovascular biomarkers in diseases such as obesity [27], T2DM [28], and CVD [29]. Prior meta-analyses have also shown that foods rich in anthocyanins can improve certain components of MetS, including total cholesterol (TC), TGs, and Low-Density Lipoprotein Cholesterol (LDL-C), although no significant changes were observed in fasting blood glucose (FBG), Homeostatic Model Assessment of Insulin Resistance (HOMA-IR), systolic blood pressure (SBP), or diastolic blood pressure (DBP) [22]. While one study reported similar outcomes, it presented contradictory results regarding certain lipid biomarkers [30]. Notably, these meta-analyses omitted four new randomized controlled trials [31–34] on anthropometric parameters, physiological measurements, and metabolic indices, and they did not fully integrate the risk factors for MetS in their systematic evaluations.

Importantly, not only is MetS associated with an increased risk of various diseases [35], but the risk of these diseases significantly escalates with the accumulation or worsening of MetS components [9, 36]. Therefore, improving the five fundamental components that constitute the diagnostic criteria for MetS is crucial for addressing MetS and its associated diseases. Recent meta-analyses have found that cranberries have limited effects on reducing human blood glucose and lipid levels [37]. Additionally, while a study has reported that foods rich in anthocyanins can significantly lower certain lipid levels in patients with MetS, they have not had a significant effect on blood pressure levels [22]. However, an RCT demonstrated that blueberries rich in anthocyanins could significantly reduce blood pressure levels in obese patients with MetS or those with stage 1 hypertension [38, 39]. Moreover, a study systematically reviewed the therapeutic roles of anthocyanins in chronic diseases, including maintaining glucose homeostasis, decreasing obesity, and protecting the liver, heart, and blood vessels [40]. Yet, these studies’ results have been inconsistent, and the overall conclusions remain controversial. To our knowledge, no previous studies have systematically evaluated the impact of dietary anthocyanins on the risk factors for MetS. Therefore, this study aims to conduct a meta-analysis of published RCTs to further assess the impact of anthocyanins on patients with risk factors for MetS.

## Materials and methods

This study was conducted and reported according to the Preferred Reporting Items for Systematic Reviews and Meta-Analyses (PRISMA) guidelines and the protocol was registered with the PROSPERO international prospective register of systematic reviews (CRD42023448429) [41].

### Study strategy

The literature search was completed on April 25, 2024, covering five electronic databases: PubMed, Web of Science, Scopus, Cochrane Library, and Embase, without language restrictions. Due to the limited number of RCTs reporting MetS events identified in preliminary searches, RCTs involving MetS risk factors were also included. Search terms were formulated by combining keywords related to anthocyanins or foods rich in anthocyanins with terms denoting risk factors for metabolic syndrome, such as abdominal obesity, elevated serum triglycerides, high blood pressure, reduced HDL cholesterol, and hyperglycemia. Reference lists of retrieved articles were reviewed to ensure no relevant studies were omitted. Unpublished studies were not included. Literature retrieval was independently conducted by two researchers (JP and JL). S1 File displays the complete search strategy and results for each database.

### Study eligibility

Relevant studies were identified using the PICOS criteria: participants, intervention, comparator, outcomes, and study design:

- Participants: Adults with MetS risk factors, with no restrictions on gender or ethnicity.
- Intervention: The interventions included the use of purified anthocyanins or anthocyanin-rich foods, which were administered in freeze-dried, powdered, and extracted forms, as well as their derivatives.
- Comparator: Control groups received either a placebo or no anthocyanins, with the main distinction being the administration of anthocyanins to the intervention group.
- Outcomes: Studies needed to provide sufficient data to calculate changes in anthropometric, physiological, and metabolic markers associated with MetS, both pre- and post-intervention, including weight, BMI, waist circumference (WC), TGs, HDL-C, LDL-C, TC, SBP, DBP, FBG, HOMA-IR, insulin, and glycated hemoglobin (HbA1c).
- Study Design: RCTs employing parallel or crossover designs.

Studies were excluded based on the following criteria:(a) Letters, comments, reviews, in vitro and animal studies, studies lacking necessary outcome measures, studies irrelevant to the topic, studies not available from clinical trial registries, or not retrievable. (b) Non-RCTs. (c) Duplicate studies. (d) Insufficient data provided. (e) Intervention groups where the dose of anthocyanins was not disclosed. (f) Studies that did not disclose the composition of the placebo or whether it contained anthocyanins. (g) Placebo containing anthocyanins.

### Ethical approval

Because all analyses were based on previously published studies, approval by an institutional review board was not necessary.

### Data collection

Data extraction for each trial included:

- Study specifications such as the first author’s surname, publication date, study design, trial location, sample size (control and intervention groups), daily dose and form of anthocyanins, duration of intervention, and funding sources.
- Participant characteristics such as average age, health status, dosage frequency, and physical activity levels.
- Health status evaluation where participants not previously diagnosed with MetS were assessed against the updated International Diabetes Federation Guidelines from 2020 [42]. Participants were classified as having MetS if they met at least three of the following five criteria [43]:
  - Waist circumference >80 cm
  - Triglycerides (TGs) levels >150 mg/dL
  - High-density lipoprotein cholesterol (HDL) <40/50 mg/dL
  - Blood pressure >130/85 mmHg
  - Fasting blood glucose >100 mg/dL

Detailed diagnostic criteria and assessment methods are provided in S1 Table.

While BMI is not a requisite for MetS diagnosis, a BMI over 25 has been shown to correlate with systemic consequences similar to those of MetS [44]. As waist circumference measurements might not be reported in most studies, our estimates considered participants with a BMI >27 likely to have a waist circumference exceeding 80 cm [30].

(d) Reported outcomes including anthropometric, physiological, and metabolic markers as previously listed. Data were standardized to common units where different units were reported.

### Quality assessment of the risk of bias

The quality of included RCTs was assessed using the **Cochrane Collaboration’s Risk of Bias Tool (ROB 1) [45]**, which evaluates several domains, including random sequence generation, allocation concealment, blinding of participants and personnel, blinding of outcome assessment, incomplete outcome data, selective outcome reporting, and other potential sources of bias. Each domain was classified as low, uncertain, or high risk of bias. The overall quality of each RCT was rated as poor, fair, or good, following procedures from previous studies. The studies were independently assessed by two investigators (JP and JL), and any disagreements were resolved through the involvement of a third investigator (LJ) to reach a consensus.

### Data synthesis and statistical analysis

Data were pooled using the weighted mean difference (WMD) with 95% confidence intervals (CI). For RCTs, crossover studies were treated as parallel designs with each intervention phase considered as an independent part of a parallel study. Control participant numbers were split in studies with multiple treatment groups and a single control group. Where mean differences (MD) and standard deviations (SD) were not reported, they were calculated as follows:

$$MD={Mean}_{post-treatment}-{Mean}_{pre-treatment}$$ (1)

SD of MD was calculated using:

$$SD=\sqrt{\left[{\left({SD}_{pre-treatment}\right)}^{2}+{\left({SD}_{post-treatment}\right)}^{2}-(2R\times {SD}_{pre-treatment}\times {SD}_{post-treatment})\right]}$$ (2)

[46], setting R (correlation coefficient) conservatively at 0.50 [47].

Standard deviations were estimated from standard errors of the mean (SEM) using:

$$SD=SEM\times \sqrt{\left(n\right)}$$ (3)

where "n" is the number of subjects.

Heterogeneity was assessed using Cochran’s Q test and I^2^ statistics [48]. Significant heterogeneity was considered when the p-value of Q statistics was less than 0.1 and I^2^ was greater than 50% [48, 49]. A fixed-effect model was applied for low heterogeneity (I^2^ < 50%), whereas a random-effects model was employed when heterogeneity was substantial (I^2^ > 50%). Given the presence of significant heterogeneity, sensitivity analysis was conducted using the One Study Removed approach to evaluate the potential influence of excluding any individual RCT on the overall results. Furthermore, for outcomes with significant heterogeneity encompassing more than five studies, subgroup analyses were performed to explore potential sources of heterogeneity associated with treatment duration, anthocyanin dosage, participant characteristics, supplement formulation, dosage frequency, and physical activity levels. Additionally, baseline risk profiles related to MetS diagnostic criteria were examined, e.g., for HDL-C meta-analysis, subgroups with high (> 1.29 mmol/L) and low (≤ 1.29 mmol/L) baseline levels were explored. In instances where more than ten studies were available, assessment of publication bias was conducted through visual inspection of the funnel plots and application of Egger’s test [50, 51]. P values <0.05 were considered significant. When publication bias was detected, results were adjusted and validated using the trim and fill method. Statistical analyses were conducted using Stata/MP 16.0 software.

## Results

### Literature search

**Fig 1:**
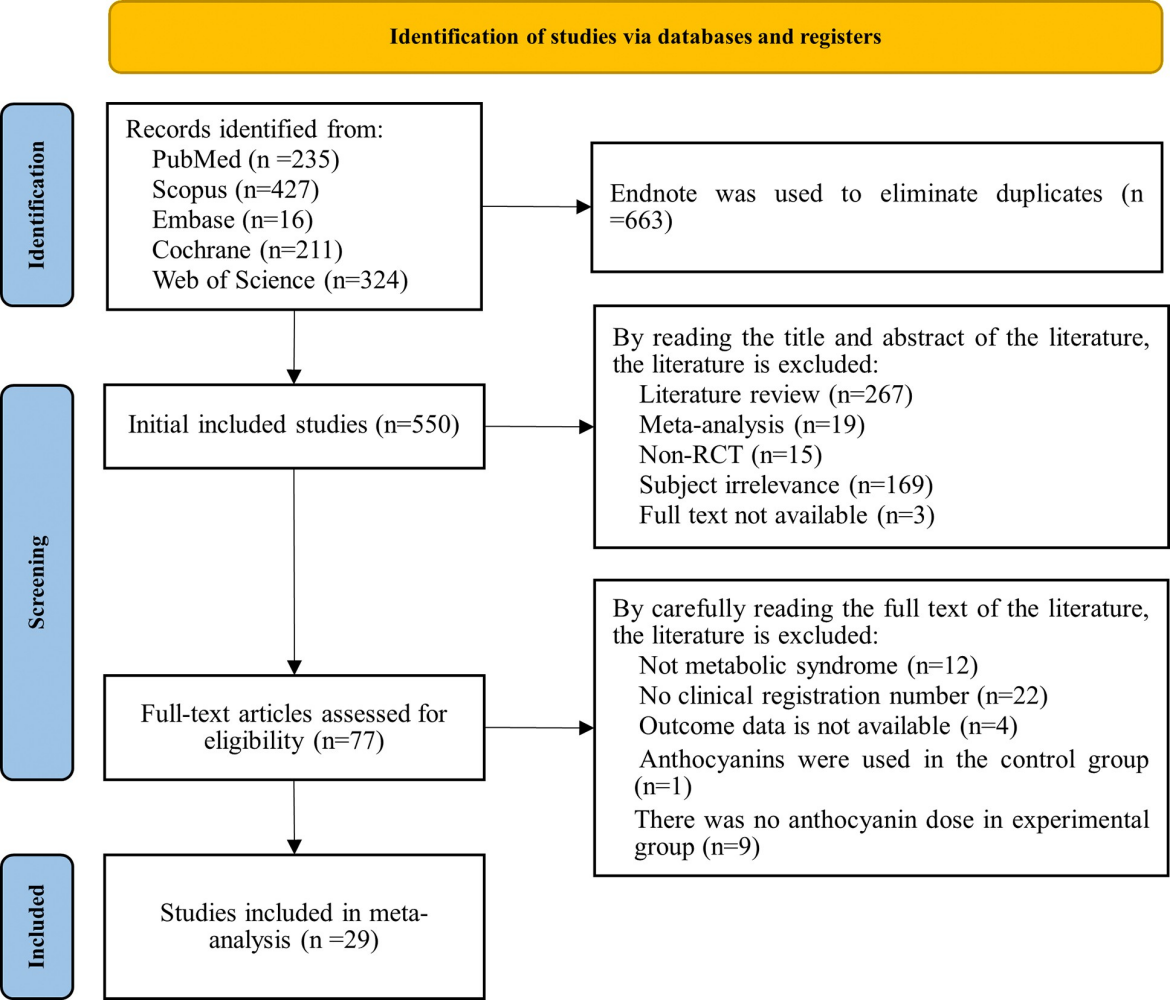
The flow chart of study selection process. Study selection flow diagram for included trials in the systematic review.

A total of 1213 records were identified through database searches. After removing 663 duplicate records, 473 records were excluded based on title and abstract screening. Full texts of the remaining 77 studies were then assessed, resulting in the exclusion of 48 studies according to the inclusion and exclusion criteria. Ultimately, 29 RCTs met the eligibility criteria and were included in this systematic review and meta-analysis. A detailed summary of the study selection process, including reasons for exclusion at each stage, is provided in S2 Table. The detailed literature screening process is illustrated in (Fig 1).

### Characteristics of studies

The 29 randomized controlled trials included in this review were published between 2010 and 2023, involving a total of 2006 participants with risk factors for MetS. All studies targeted adults, with participants’ mean ages ranging from 28.7 to 68 years. Among the 29 studies, 8 were conducted in China [52–59], 5 in the United States [39, 60–63], 3 in Australia [33, 64, 65], 3 in Iran [66–68], 3 in the United Kingdom [69–71], 2 in Korea [72, 73], and 1 each in Canada [74], Denmark [31], Norway [75], Sweden [76], and a multicenter study conducted in Europe [77]. Of these, 9 studies focused on interventions involving purified anthocyanin compounds or anthocyanin capsule supplementation [52–59, 75], 11 studies investigated the supplementation of anthocyanin-rich berries and their derivatives (fresh/freeze-dried) [31, 39, 60–63, 66, 68–70, 76], 4 studies explored the effects of anthocyanin-rich fruit juice from various fruits [33, 65, 71, 77], and 6 studies examined anthocyanin-rich foods and their derivatives [64, 67, 69, 72–74]. The duration of the interventions ranged from 4 days to 24 weeks, with anthocyanin doses varying from a minimum of 0.26 mg/day to a maximum of 900 mg/day. Detailed characteristics of the included studies are provided in S3 Table. The basic characteristics of the included studies are presented in Table 1, with additional detailed information provided in S4 Table.

**Table 1:** The basic characteristics of the included studies.

No.	Study	Blind method	Location	Health status	Sample size (I/C)	Age	Duration	Intervention	Anthocyanin dosage (mg/d)	Dosage frequency	Physical activity levels	Outcome
1	Ah Jin Jung2021	Double blind	Korea	Postmenopausal women with obesity	46/40	45–69	12wk	Black rice extract	327.6mg/d	Twice a day	N/A	weight、BMI、LDL-C、HDL-C、TG、TC、SBP、DBP、FBG、insulin
2	April J Stull2010	Double blind	America	Obese/Insulin resistant	15/17	≥20	6wk	Smoothie with blueberry bioactives	668mg/d	Twice a day	N/A	weight、BMI、LDL-C、HDL-C、TG、TC、SBP、DBP、FBG、insulin
3	Arpita Basu2014	N/A	America	MetS	15/15	49±10	12wk	Freeze-dried strawberries	a: 78mg/d b: 155mg/d	Twice a day	maintain usual physical activity	weight、BMI、WC、LDL-C、HDL-C、TG、TC、SBP、DBP、HOMA-IR、insulin、HbA1c
4	Christine B2023	Triple-blinded	Denmark	Type 2 Diabetes	23/23	30–80	8wk	Aronia berry pulp	a: 893mg/d b: 533mg/d	N/A	N/A	LDL-C、HDL-C、TG、TC、SBP
5	Dan Li2015	Double blind	China	Type 2 Diabetes	29/29	56–67	6months	Purified anthocyanins	320mg/d	Twice a day	maintain usual physical activity	BMI、LDL-C、HDL-C、TC、SBP、DBP、FBG、HOMA-IR、insulin、HbA1c
6	David Briskey2022	Double blind	Australia	Overweight	65/71	20–65	a: 3months b: 6months	Moro blood orange extract	3.6mg/d	Once a day	asked to undertake 30 min of walking 3 times per week	weight、BMI、WC、LDL-C、HDL-C、FBG、insulin
7	Hanyue Zhang2020	Double blind	China	Hyperlipidemia	42/43	35–70	12wk	Purified anthocyanins	a: 40mg/d b: 80mg/d c: 320mg/d	Twice a day	N/A	TC
8	Hassan Aboufarrag2022	Double blind	United Kingdom	Elevated Cholesterol	52/52	>45	28d	Black rice extract/Bilberry extract	320mg/d	Once a day	N/A	LDL-C、HDL-C、TG、TC、FBG
9	Kim S Stote2020	Double blind	America	Diabetes	26/26	45–75	a: 4wk b: 8wk	Freeze-dried blueberries	261.8mg/d	Twice a day	N/A	weight、BMI、WC、LDL-C、HDL-C、TG、TC、SBP、DBP、FBG、insulin
10	Lilith Arevström2019	Double blind	Sweden	AMI	25/25	≥18	8wk	Bilberry powder	900mg/d	Three times a day	N/A	weight、BMI、SBP、DBP
11	Liping Yang2017	Double blind	China	Diabetes	80/80	40–75	12wk	Purified anthocyanins	320mg/d	Twice a day	N/A	LDL-C、HDL-C、TG、TC、FBG、HOMA-IR、insulin、HbA1c
12	Liping Yang2020	Double blind	China	Elevated fasting glucose	54/50	61.27±8.36/60.88±6.97	12wk	Purified anthocyanins	320mg/d	N/A	N/A	FBG、HOMA-IR
13	Liping Yang2021	Double blind	China	Prediabetes or newly diagnosed diabetes	77/62	40–75	12wk	Purified anthocyanins	320mg/d	Twice a day	maintain usual physical activity	weight、BMI、WC、SBP、DBP、FBG、HOMA-IR
14	Lu Li2020	Single-blind	European	Overweight and obese	15/15	20–45	4wk	Blood orange juice	9.6±0.52mg/d	Twice a day	N/A	LDL-C、HDL-C、TG、TC、SBP、DBP
15	Myoungsook Lee2016	Double blind	Korea	Overweight/Obese	40/40	19–65	8wk	Black soybean testa extract	31.45mg/d	Three times a day	maintain usual physical activity	weight、BMI、WC、LDL-C、HDL-C、TG、TC、SBP
16	Olivia R L Wright2013	Double blind	Australia	overweight and obese	8/8	18–65	4wk	Dried purple carrot	118.5mg/d	Three times a day	maintain usual physical activity	weight、WC、LDL-C、HDL-C、TC、SBP、DBP
17	Pei-Wen Zhang2015	Double blind	China	NAFLD	34/29	44.9±7.5/46.9±7.7	12wk	Purified anthocyanins	320mg/d	Twice a day	N/A	weight、BMI、WC、SBP、DBP
18	Reza Amani2014	Double blind	Iran	T2DM	19/17	35–60	6wk	Freeze-dried strawberry beverage	154mg/d	Twice a day	maintain usual physical activity	LDL-C、HDL-C、TG、TC、SBP、DBP
19	Reza Mohtashami2019	Double blind	Iran	MetS	50/50	40–80	2months	Vaccinium arctostaphylos leaf extract	0.2583±0.00175mg/d	N/A	N/A	LDL-C、HDL-C、TG、TC、FBG、HbA1c
20	S.Kianbakht2014	Double blind	Iran	Hyperlipidemic	40/40	20–60	2months	Whortleberry extract	7.35mg/d	Three times a day	maintain usual physical activity	weight、LDL-C、HDL-C、TG、TC
21	Sarah A Johnson2020	Single-blind	America	MetS	9/10	20–60	a: 6wk b: 12wk	Montmorency Tart Cherry Juice	176mg/d	Twice a day	maintain usual physical activity	weight、BMI、WC、LDL-C、HDL-C、TG、SBP、DBP、FBG、HOMA-IR、insulin
22	Sarah A.Johnson2015	Double blind	America	Hypertension	20/20	45–65	a: 4wk b: 8wk	Free-dried blueberry powder	103.18mg/d	Twice a day	N/A	weight、BMI、WC、SBP、DBP
23	SS Hassellund2012	Double blind	Norway	Hypertension	27/27	35–51	4wk	Purified anthocyanin	640mg/d	Twice a day	N/A	SBP、DBP
24	Tamer H Gamel2020	Single-blind	Canada	overweight and obese	17/16	18–70	a: 4wk b: 8wk	Purple wheat	1.65mg/d	Four times a day	refrain from participating in strenuous physical activity	weight、LDL-C、HDL-C、TC、SBP、DBP、FBG、insulin
25	Terun Desai12020	Single-blind	United Kingdom	MetS	12/12	28–62	7d	Montmorency tart cherry juice	270mg/d	Once a day	N/A	LDL-C、HDL-C、TG、TC、SBP、DBP、FBG、insulin
26	Vinicius A do Rosario2020	Double blind	Australia	Overweight	16/16	>55	4d	Queen garnet plum juice	200.86mg/d	Once a day	N/A	SBP、DBP
27	Wendy J2018	Open label	United Kingdom	Overweight and obese	41/41	25–84	28d	Blood orange juice	50mg/d	Twice a day	N/A	LDL-C、HDL-C、TC、SBP、SBP、DBP
28	Xiandan Zhang2016	Double blind	China	Hypercholesterolemia	73/73	40–65	a: 12wk b: 24wk	Purified anthocyanins	320mg/d	Twice a day	maintain usual physical activity	weight、BMI、LDL-C、HDL-C、TC、SBP、DBP
29	Zhongliang Xu2021	Double blind	China	Dyslipidemia	43/46	35–70	12wk	Purified anthocyanins	a: 40mg/d b: 80mg/d c: 320mg/d	Twice a day	maintain usual physical activity	LDL-C、HDL-C、TG、TC

If the article does not report information on the blinding method, dosage frequency, funding, and physical activity levels, it is marked as ‘Not available (N/A)’.

Abbreviations: I—Intervention group, C—Control group; BMI—Body mass index; WC—Waist circumference; HDL-C—High-density lipoprotein cholesterol; LDL-C—Low-density lipoprotein cholesterol; TGs—Triglycerides; TC—Total cholesterol; FBG—Fasting blood glucose; HbA1c - Glycated hemoglobin; HOMA-IR—Homeostatic Model Assessment of Insulin Resistance.

### Risk of bias evaluation of studies and overall estimates

**Fig 2:**
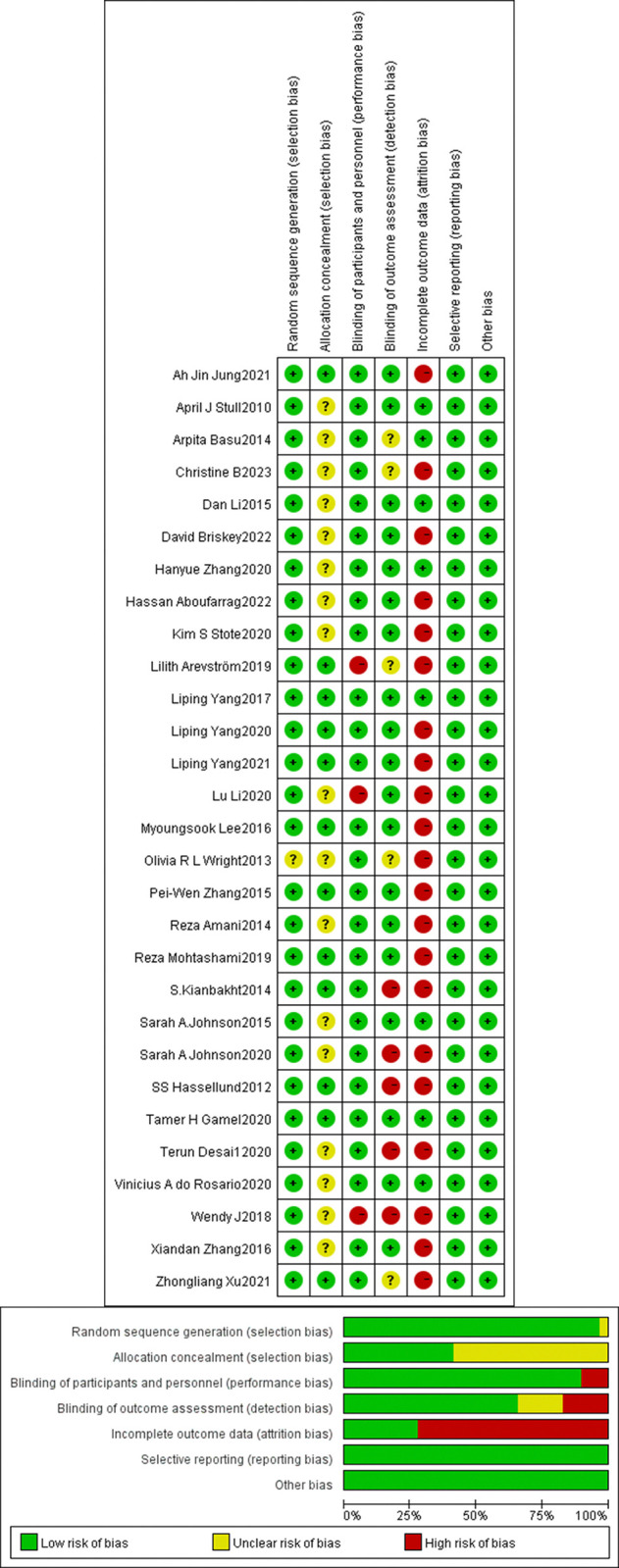
Assessment of study bias in the included research.

The risk of bias assessment is presented in (Fig 2). Detailed results of the risk of bias assessment are provided in S5 Table. Of the 29 included studies, all except one [64] reported using random allocation. Twelve studies described the allocation concealment process [54–57, 59, 67, 68, 72–76], thus being assessed as low risk for allocation concealment, while the remaining 17 studies were rated as unclear [31, 33, 39, 52, 53, 58, 60–66, 69–71, 77]. Three studies were open-label [71, 76, 77], and thus assessed as high risk for blinding of participants and personnel, whereas the rest implemented blinding and were rated as low risk. For blinding of outcome assessors, 5 studies did not perform adequate blinding [63, 68, 70, 71, 75], and were therefore considered high risk in this domain; 5 studies did not describe blinding in this domain [31, 59, 61, 64, 76], resulting in an unclear risk of bias; the remaining studies were rated as low risk. Twenty-one studies experienced dropouts during the trial and follow-up [31, 33, 55–59, 62–64, 66–73, 75–77], leading to a high risk assessment for incomplete outcome data. All randomized controlled trials were assessed as having low risk of bias in terms of selective reporting and other biases.

### Dietary anthocyanins consumption and central obesity

**Fig 3:**
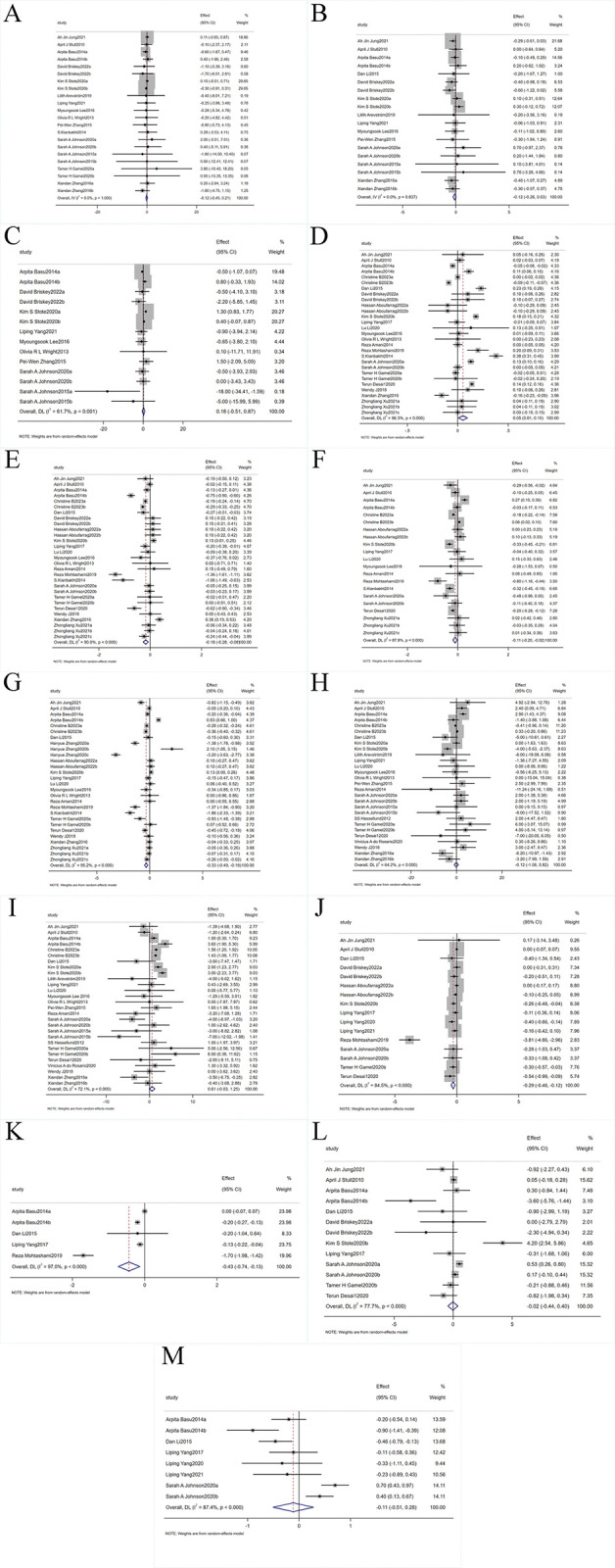
Forest plot. Forest plot detailing WMD and 95% CIs for the effect of dietary anthocyanin consumption on (A) Weight (kg), (B) BMI (kg/m^2^), (C) WC (cm), (D) HDL-C (mmol/L), (E) LDL-C (mmol/L), (F) TGs (mmol/L), (G) TC (mmol/L), (H) SBP (mmHg), (I) DBP (mmHg), (J) FBG (mmol/L), (K) HbA1c (%), (L) insulin (mU/L), and (M) HOMA-IR.

13 eligible articles [33, 39, 56–58, 60–64, 72, 73, 76] with 19 treatment arms, involving a total of 1347 participants, showed no statistically significant heterogeneity in the weight data (p = 1.000, I^2^ = 0%). Therefore, a fixed-effects model was used. The results indicated no significant change in weight levels in the dietary anthocyanin supplementation group compared to the control group (WMD: -0.12 kg, 95% CI: -0.45 to 0.21, p = 0.473) (Fig 3A). Sensitivity analysis showed that removing any single study did not significantly affect the overall effect size.

Among the eligible articles, 13 studies [33, 39, 52, 56–58, 60–63, 72, 73, 76] with 19 treatment arms, involving 1347 participants, reported BMI data with no significant heterogeneity (p = 0.837, I^2^ = 0%). Thus, a fixed-effects model was used. The results indicated that the impact of dietary anthocyanins on BMI was negligible compared to the control group (WMD: -0.12 kg/m^2^, 95% CI: -0.26 to 0.03, p = 0.12) (Fig 3B). Sensitivity analysis revealed that by excluding the study by Kim S Stote 2020b [62], the non-significant effect of dietary anthocyanins on BMI became significant (WMD: -0.17 kg/m^2^, 95% CI: -0.33 to -0.02, p < 0.05).

9 studies [33, 39, 56, 57, 61–64, 73] with 14 treatment arms, involving 845 participants, revealed significant heterogeneity in WC outcomes (p = 0.001, I^2^ = 61.7%). Using a random-effects model, the data indicated that dietary anthocyanins had no significant effect on WC (WMD: 0.18 cm, 95% CI: -0.51 to 0.87, p = 0.613) (Fig 3C). Sensitivity analysis showed no significant difference in WC results when any single study was removed. Subgroup analysis revealed a significant reduction in WC when the intervention dose was categorized as ≤ 100 mg/day (WMD: -0.55 cm, 95% CI: -1.09 to -0.01, p = 0.047) (Table 2). However, subgroup analyses based on intervention duration, anthocyanin dosage, formula, health status, dosage frequency, physical activity levels, and baseline WC revealed no significant changes. Detailed results, including effect sizes and heterogeneity analyses for WC and other health outcomes, are provided in S6 Table.

**Table 2:** Summary of subgroup analysis results.

Outcome	Variable	Comparisons	Effect size	Lower	Upper	P value	I^2^ (%)	Q-statistics (P)	P _between subgroups_
WC	Overall effect	14	0.18	-0.51	0.87	0.613	61.7%	0.001	
	Intervention duration								
	≤ 4 weeks	3	-2.75	-11.79	6.28	0.550	62.6%	0.069	0.256
	4 < duration ≤ 8 weeks	4	0.34	-0.12	0.81	0.145	0.0%	0.609	
	8 < duration ≤ 12 weeks	6	-0.18	-0.72	0.35	0.503	3.4%	0.395	
	> 12 weeks	1	-2.20	-5.85	1.45	0.237	-	-	
	Anthocyanins dosage								
	≤ 100 mg/day	4	-0.55	-1.09	-0.01	0.047*	0.0%	0.836	0.024
	100 < dose ≤ 300 mg/day	8	0.69	-0.02	1.40	0.055	49.7%	0.053	
	300 < dose ≤ 500 mg/day	2	0.10	-2.22	2.42	0.930	0.0%	0.317	
	> 500 mg/day	-	-	-	-	-	-	-	
	Health status								
	Obesity and Overweight	4	-1.10	-3.00	0.81	0.260	0.0%	0.914	0.145
	Insulin Resistance and Diabetes Mellitus	3	0.73	-0.12	1.58	0.094	75.6%	0.016	
	Hypertension	2	-10.01	-22.41	2.39	0.114	39.9%	0.197	
	Dyslipidemia	-	-	-	-	-	-	-	
	Metabolic Syndrome	4	-0.08	-0.86	0.71	0.852	26.8%	0.251	
	Other	1	1.50	-2.09	5.09	0.413	-	-	
	Formula								
	Purified anthocyanins	2	0.10	-2.22	2.42	0.930	0.0%	0.317	0.587
	Anthocyanins-rich berries and their derivatives	8	0.34	-0.47	1.16	0.406	76.4%	0.000	
	Anthocyanin-rich fruit juices from other fruits	2	-1.34	-3.90	1.22	0.305	0.0%	0.515	
	Anthocyanin-rich foods and their derivatives	2	-0.79	-3.65	2.07	0.586	0.0%	0.878	
	Dosage frequency								
	Once a day	2	-1.34	-3.90	1.22	0.305	0.0%	0.515	0.379
	Twice a day	10	0.33	-0.42	1.09	0.390	70.8%	0.000	
	Three times a day	2	-0.79	-3.65	2.07	0.586	0.0%	0.878	
	Four times a day	-	-	-	-	-	-	-	
	N/A	-	-	-	-	-	-	-	
	Physical activity levels								
	Maintain usual physical activity	7	-0.27	-0.76	0.21	0.267	0.0%	0.619	0.119
	Required to exercise a specific amount	2	-1.34	-3.90	1.22	0.305	0.0%	0.515	
	Avoid strenuous physical activity	-	-	-	-	-	-	-	
	N/A	5	0.78	-0.24	1.79	0.134	69.8%	0.010	
	Baseline								
	> 102 cm	9	0.31	-0.41	1.02	0.404	69.5%	0.001	0.402
	≤ 102 cm	5	-0.82	-3.37	1.72	0.525	36.6%	0.177	
HDL-C	Overall effect	29	0.05	0.01	0.10	0.026**	96.3%	0.000	
	Intervention duration								
	≤ 4 weeks	7	0.02	-0.08	0.13	0.649	92.1%	0.000	0.655
	4 < duration ≤ 8 weeks	10	0.08	0.00	0.16	0.04*	98.0%	0.000	
	8 < duration ≤ 12 weeks	9	0.02	-0.03	0.08	0.397	76.7%	0.000	
	> 12 weeks	3	0.06	-0.23	0.34	0.703	97.3%	0.000	
	Anthocyanins dosage								
	≤ 100 mg/day	12	0.08	0.00	0.17	0.061	92.8%	0.000	0.046
	100 < dose ≤ 300 mg/day	7	0.09	0.04	0.14	0.000*	92.2%	0.000	
	300 < dose ≤ 500 mg/day	7	-0.01	-0.16	0.14	0.901	93.0%	0.000	
	> 500 mg/day	3	-0.03	-0.10	0.05	0.489	96.6%	0.000	
	Health status								
	Obesity and Overweight	10	0.00	-0.02	0.03	0.795	0.0%	0.641	0.248
	Insulin Resistance and Diabetes Mellitus	7	0.05	-0.04	0.13	0.283	98.2%	0.000	
	Hypertension	-	-	-	-	-	-	-	
	Dyslipidemia	7	0.02	-0.19	0.22	0.865	95.2%	0.000	
	Metabolic Syndrome	6	0.08	0.00	0.17	0.042*	97.1%	0.000	
	Other	-	-	-	-	-	-	-	
	Formula								
	Purified anthocyanins	6	0.02	-0.13	0.18	0.757	93.8%	0.000	0.618
	Anthocyanins-rich berries and their derivatives	12	0.06	0.00	0.13	0.055	98.3%	0.000	
	Anthocyanin-rich fruit juices from other fruits	4	0.10	0.01	0.19	0.029*	0.0%	0.999	
	Anthocyanin-rich foods and their derivatives	7	0.03	-0.05	0.10	0.512	63.0%	0.013	
	Dosage frequency								
	Once a day	5	0.05	-0.05	0.16	0.335	67.0%	0.016	0.220
	Twice a day	16	0.05	-0.01	0.11	0.112	94.3%	0.000	
	Three times a day	3	0.14	-0.15	0.43	0.352	95.3%	0.000	
	Four times a day	2	-0.02	-0.05	0.01	0.245	0.0%	1.000	
	N/A	3	0.02	-0.07	0.10	0.737	97.2%	0.000	
	Physical activity levels								
	Maintain usual physical activity	13	0.06	-0.02	0.14	0.117	95.5%	0.000	0.050
	Required to exercise a specific amount	2	0.10	-0.02	0.22	0.092	0.0%	1.000	
	Avoid strenuous physical activity	2	-0.02	-0.05	0.01	0.245	0.0%	1.000	
	N/A	12	0.05	-0.03	0.12	0.238	97.6%	0.000	
	Baseline								
	> 1.29 mmol/L	16	0.03	-0.03	0.09	0.285	84.6%	0.000	0.343
	≤ 1.29 mmol/L	13	0.07	0.01	0.14	0.036*	97.9%	0.000	
LDL-C	Overall effect	29	-0.18	-0.28	-0.08	0.000**	90.0%	0.000	
	Intervention duration								
	≤ 4 weeks	7	-0.10	-0.33	0.14	0.427	64.2%	0.010	0.281
	4 < duration ≤ 8 weeks	10	-0.30	-0.46	-0.13	0.000*	94.1%	0.000	
	8 < duration ≤ 12 weeks	9	-0.18	-0.36	0.00	0.052	86.3%	0.000	
	> 12 weeks	3	0.07	-0.34	0.47	0.739	89.1%	0.000	
	Anthocyanins dosage								
	≤ 100 mg/day	12	-0.25	-0.51	0.02	0.066	90.0%	0.000	0.648
	100 < dose ≤ 300 mg/day	7	-0.19	-0.52	0.14	0.255	93.6%	0.000	
	300 < dose ≤ 500 mg/day	7	-0.05	-0.26	0.16	0.654	82.9%	0.000	
	> 500 mg/day	3	-0.18	-0.30	-0.07	0.001*	89.9%	0.000	
	Health status								
	Obesity and Overweight	10	-0.04	-0.13	0.05	0.415	0.0%	0.797	0.117
	Insulin Resistance and Diabetes Mellitus	7	-0.13	-0.25	-0.01	0.032*	89.4%	0.000	
	Hypertension	-	-	-	-	-	-	-	
	Dyslipidemia	7	-0.09	-0.36	0.17	0.497	87.6%	0.000	
	Metabolic Syndrome	6	-0.48	-0.86	-0.11	0.012*	95.7%	0.000	
	Other	-	-	-	-	-	-	-	
	Formula								
	Purified anthocyanins	6	-0.07	-0.28	0.14	0.520	84.8%	0.000	0.109
	Anthocyanins-rich berries and their derivatives	12	-0.22	-0.34	-0.09	0.001*	92.0%	0.000	
	Anthocyanin-rich fruit juices from other fruits	4	0.03	-0.14	0.19	0.756	0.0%	0.786	
	Anthocyanin-rich foods and their derivatives	7	-0.28	-0.78	-0.22	0.270	91.5%	0.000	
	Dosage frequency								
	Once a day	5	-0.05	-0.35	0.25	0.748	79.5%	0.001	0.014
	Twice a day	16	-0.10	-0.24	0.04	0.169	87.6%	0.000	
	Three times a day	3	-0.52	-1.10	0.07	0.082	76.6%	0.014	
	Four times a day	2	-0.01	-0.36	0.34	0.954	0.0%	0.956	
	N/A	3	-0.53	-0.77	-0.29	0.000*	97.6%	0.000	
	Physical activity levels								
	Maintain usual physical activity	13	-0.19	-0.39	0.01	0.060	89.9%	0.000	0.084
	Required to exercise a specific amount	2	0.10	-0.12	0.32	0.375	0.0%	1.000	
	Avoid strenuous physical activity	2	-0.01	-0.36	0.34	0.954	0.0%	0.956	
	N/A	12	-0.22	-0.36	-0.08	0.002*	92.7%	0.000	
TGs	Overall effect	21	-0.11	-0.20	-0.02	0.021**	87.9%	0.000	
	Intervention duration								
	≤ 4 weeks	4	-0.04	-0.22	0.15	0.718	67.0%	0.028	0.091
	4 < duration ≤ 8 weeks	9	-0.23	-0.38	-0.08	0.002*	93.1%	0.000	
	8 < duration ≤ 12 weeks	8	-0.01	-0.16	0.14	0.897	68.2%	0.003	
	> 12 weeks	-	-	-	-	-	-	-	
	Anthocyanins dosage								
	≤ 100 mg/day	7	-0.13	-0.45	0.19	0.439	90.2%	0.000	0.421
	100 < dose ≤ 300 mg/day	6	-0.19	-0.31	-0.07	0.002*	60.6%	0.026	
	300 < dose ≤ 500 mg/day	5	-0.04	-0.18	0.10	0.588	18.7%	0.295	
	> 500 mg/day	3	-0.07	-0.26	0.12	0.451	97.0%	0.000	
	Health status								
	Obesity and Overweight	4	-0.13	-0.25	0.00	0.046*	0.0%	0.414	0.882
	Insulin Resistance and Diabetes Mellitus	6	-0.11	-0.27	0.04	0.155	94.1%	0.000	
	Hypertension	-							
	Dyslipidemia	6	-0.06	-0.24	0.12	0.514	65.4%	0.013	
	Metabolic Syndrome	6	-0.18	-0.42	0.06	0.151	91.7%	0.000	
	Other								
	Formula								
	Purified anthocyanins	4	-0.01	-0.19	0.17	0.880	0.0%	0.995	0.364
	Anthocyanins-rich berries and their derivatives	12	-0.10	-0.21	0.01	0.085	92.4%	0.000	
	Anthocyanin-rich fruit juices from other fruits	1	0.15	-0.33	0.63	0.543	-	-	
	Anthocyanin-rich foods and their derivatives	4	-0.34	-0.72	0.05	0.087	77.7%	0.004	
	Dosage frequency								
	Once a day	3	-0.06	-0.26	0.14	0.578	73.8%	0.022	0.042
	Twice a day	13	-0.07	-0.21	0.07	0.341	78.7%	0.000	
	Three times a day	2	-0.32	-0.45	-0.19	0.000*	0.0%	0.950	
	Four times a day	-	-	-	-	-	-	-	
	N/A	3	-0.21	-0.44	0.02	0.075	97.6%	0.000	
	Physical activity levels								
	Maintain usual physical activity	10	-0.06	-0.25	0.13	0.521	80.8%	0.000	0.459
	Required to exercise a specific amount	-	-	-	-	-	-	-	
	Avoid strenuous physical activity	-	-	-	-	-	-	-	
	N/A	11	-0.14	-0.26	-0.03	0.014*	91.3%	0.000	
	Baseline								
	> 1.7 mmol/L	10	-0.19	-0.34	-0.04	0.013*	68.5%	0.001	0.161
	≤ 1.7 mmol/L	11	-0.05	-0.17	0.06	0.355	91.4%	0.000	
TC	Overall effect	28	-0.34	-0.49	-0.18	0.000**	95.2%	0.000	
	Intervention duration								
	≤ 4 weeks	7	-0.17	-0.45	0.10	0.216	63.9%	0.011	0.254
	4 < duration ≤ 8 weeks	9	-0.36	-0.52	-0.19	0.000*	93.7%	0.000	
	8 < duration ≤ 12 weeks	10	-0.37	-0.93	0.20	0.207	97.7%	0.000	
	> 12 weeks	2	-0.07	-0.32	0.17	0.560	0.0%	0.686	
	Anthocyanins dosage								
	≤ 100 mg/day	12	-0.41	-0.78	-0.04	0.032*	91.3%	0.000	0.294
	100 < dose ≤ 300 mg/day	5	0.12	-0.37	0.61	0.630	94.7%	0.000	
	300 < dose ≤ 500 mg/day	8	-0.55	-1.17	0.08	0.085	96.4%	0.000	
	> 500 mg/day	3	-0.26	-0.36	-0.15	0.000*	89.7%	0.000	
	Health status								
	Obesity and Overweight	8	-0.28	-0.57	0.01	0.060	74.3%	0.000	0.619
	Insulin Resistance and Diabetes Mellitus	7	-0.15	-0.28	-0.02	0.026*	90.8%	0.000	
	Hypertension	-	-	-	-	-	-	-	
	Dyslipidemia	10	-0.50	-1.11	0.11	0.107	96.8%	0.000	
	Metabolic Syndrome	4	-0.28	-1.04	0.49	0.481	97.8%	0.000	
	Other	-	-	-	-	-	-	-	
	Formula								
	Purified anthocyanins	9	-0.41	-1.02	0.19	0.181	96.5%	0.000	0.321
	Anthocyanins-rich berries and their derivatives	10	-0.16	-0.36	0.03	0.094	96.8%	0.000	
	Anthocyanin-rich fruit juices from other fruits	2	-0.02	-0.34	0.31	0.910	0.0%	0.630	
	Anthocyanin-rich foods and their derivatives	7	-0.49	-0.93	-0.06	0.027*	82.3%	0.000	
	Dosage frequency								
	Once a day	3	-0.10	-0.50	0.29	0.610	75.7%	0.016	0.570
	Twice a day	17	-0.24	-0.55	0.08	0.141	96.0%	0.000	
	Three times a day	3	-0.76	-1.95	0.42	0.206	91.8%	0.000	
	Four times a day	2	-0.44	-1.42	0.54	0.383	82.8%	0.016	
	N/A	3	-0.40	-0.53	-0.26	0.000*	92.2%	0.000	
	Physical activity levels								
	Maintain usual physical activity	11	-0.18	-0.54	0.18	0.318	93.9%	0.000	0.446
	Required to exercise a specific amount	-	-	-	-	-	-	-	
	Avoid strenuous physical activity	2	-0.44	-1.42	0.54	0.383	82.8%	0.016	
	N/A	15	-0.44	-0.63	-0.26	0.000*	95.8%	0.000	
SBP	Overall effect	28	-0.12	-1.06	0.82	0.801	64.2%	0.000	
	Intervention duration								
	≤ 4 weeks	9	0.37	-1.05	1.79	0.609	0.0%	0.852	0.164
	4 < duration ≤ 8 weeks	10	-0.48	-1.78	0.82	0.466	76.8%	0.000	
	8 < duration ≤ 12 weeks	7	0.41	-2.11	2.93	0.750	70.8%	0.002	
	> 12 weeks	2	-3.96	-7.60	-0.32	0.033*	0.0%	0.632	
	Anthocyanins dosage								
	≤ 100 mg/day	6	2.73	1.40	4.06	0.000*	0.0%	0.797	0.001
	100 < dose ≤ 300 mg/day	11	-1.12	-3.12	0.89	0.275	61.4%	0.004	
	300 < dose ≤ 500 mg/day	6	-1.91	-5.12	1.31	0.245	49.3%	0.079	
	> 500 mg/day	5	0.23	-0.62	1.07	0.599	55.8%	0.060	
	Health status								
	Obesity and Overweight	9	2.27	0.51	4.02	0.011*	0.0%	0.943	0.005
	Insulin Resistance and Diabetes Mellitus	8	-0.71	-1.91	0.49	0.244	80.5%	0.000	
	Hypertension	3	-1.26	-7.02	4.50	0.668	32.1%	0.229	
	Dyslipidemia	2	-4.70	-8.08	-1.32	0.006*	38.4%	0.000	
	Metabolic Syndrome	5	1.20	-0.97	3.37	0.278	61.2%	0.035	
	Other	2	0.02	-7.55	7.59	0.995	36.5%	0.209	
	Formula								
	Purified anthocyanins	6	-2.15	-5.02	0.72	0.141	39.9%	0.139	0.209
	Anthocyanins-rich berries and their derivatives	14	-0.03	-1.11	1.05	0.957	77.4%	0.000	
	Anthocyanin-rich fruit juices from other fruits	3	1.67	-2.33	5.67	0.413	0.0%	0.783	
	Anthocyanin-rich foods and their derivatives	5	2.42	-1.21	6.06	0.192	0.0%	0.689	
	Dosage frequency								
	Once a day	2	-1.90	-9.05	5.26	0.603	0.0%	0.359	0.548
	Twice a day	19	-0.37	-2.00	1.27	0.660	73.2%	0.000	
	Three times a day	3	-1.39	-6.25	3.48	0.577	0.0%	0.714	
	Four times a day	2	5.01	-1.43	11.44	0.127	0.0%	0.761	
	N/A	2	-0.04	-0.76	0.69	0.924	72.5%	0.057	
	Physical activity levels								
	Maintain usual physical activity	11	-0.95	-3.14	1.24	0.395	67.8%	0.001	0.228
	Required to exercise a specific amount	-	-	-	-	-	-	-	
	Avoid strenuous physical activity	2	5.01	-1.43	11.44	0.127	0.0%	0.761	
	N/A	15	-0.22	-1.27	0.84	0.690	64.0%	0.000	
	Baseline								
	> 130 mmHg	10	-1.08	-2.90	0.74	0.244	0.0%	0.664	0.289
	≤ 130 mmHg	18	0.06	-1.01	1.13	0.912	74.7%	0.000	
DBP	Overall effect	28	0.61	-0.03	1.25	0.061	72.1%	0.000	
	Intervention duration								
	≤ 4 weeks	9	1.72	1.02	2.42	0.000*	0.0%	0.359	0.033
	4 < duration ≤ 8 weeks	10	0.33	-0.68	1.35	0.519	86.1%	0.000	
	8 < duration ≤ 12 weeks	7	0.68	-0.87	2.23	0.391	67.6%	0.005	
	> 12 weeks	2	-1.31	-3.96	1.34	0.332	0.0%	0.596	
	Anthocyanins dosage								
	≤ 100 mg/day	6	1.01	-0.20	2.22	0.102	10.9%	0.346	0.161
	100 < dose ≤ 300 mg/day	11	0.22	-1.34	1.78	0.782	78.9%	0.000	
	300 < dose ≤ 500 mg/day	6	-0.95	-2.49	0.60	0.230	17.2%	0.303	
	> 500 mg/day	5	0.91	0.14	1.68	0.020*	76.3%	0.002	
	Health status								
	Obesity and Overweight	9	-0.24	-1.58	1.10	0.725	13.0%	0.327	0.099
	Insulin Resistance and Diabetes Mellitus	8	1.27	0.55	2.00	0.001*	81.7%	0.000	
	Hypertension	3	-2.68	-7.77	2.41	0.783	73.6%	0.023	
	Dyslipidemia	2	-1.96	-5.00	1.08	0.207	42.1%	0.189	
	Metabolic Syndrome	5	0.44	-1.86	2.74	0.707	80.4%	0.000	
	Other	2	-0.76	-6.18	4.66	0.783	63.1%	0.100	
	Formula								
	Purified anthocyanins	6	-0.49	-2.14	1.16	0.560	29.6%	0.213	0.496
	Anthocyanins-rich berries and their derivatives	14	0.91	0.17	1.64	0.015*	82.2%	0.000	
	Anthocyanin-rich fruit juices from other fruits	3	0.40	-2.16	2.95	0.760	0.0%	0.900	
	Anthocyanin-rich foods and their derivatives	5	0.99	-2.12	4.10	0.531	42.7%	0.137	
	Dosage frequency								
	Once a day	2	0.32	-3.55	4.20	0.871	0.0%	0.446	0.003
	Twice a day	19	-0.06	-1.11	0.98	0.904	79.2%	0.000	
	Three times a day	3	-1.93	-5.06	1.21	0.228	0.0%	0.658	
	Four times a day	2	5.64	1.13	10.15	0.014*	0.0%	0.835	
	N/A	2	1.49	1.24	1.74	0.000*	0.0%	0.610	
	Physical activity levels								
	Maintain usual physical activity	11	-0.53	-2.09	1.04	0.508	71.2%	0.000	0.024
	Required to exercise a specific amount	-	-	-	-	-	-	-	
	Avoid strenuous physical activity	2	5.64	1.13	10.15	0.014*	0.0%	0.835	
	N/A	15	0.99	0.28	1.70	0.006*	73.0%	0.000	
	Baseline								
	> 85 mmHg	4	1.24	-0.73	3.20	0.217	74.2%	0.009	0.422
	≤ 85 mmHg	24	0.38	-0.35	1.11	0.308	72.8%	0.000	
FBG	Overall effect	16	-0.29	-0.46	-0.12	0.001**	84.5%	0.000	
	Intervention duration								
	≤ 4 weeks	3	-0.12	-0.32	0.08	0.224	58.6%	0.090	0.208
	4 < duration ≤ 8 weeks	5	-0.74	-1.27	-0.20	0.007*	95.2%	0.000	
	8 < duration ≤ 12 weeks	6	-0.18	-0.31	-0.05	0.006*	0.0%	0.473	
	> 12 weeks	2	-0.22	-0.52	0.08	0.149	0.0%	0.693	
	Anthocyanins dosage								
	≤ 100 mg/day	4	-0.95	-1.81	-0.09	0.031*	95.7%	0.000	0.001
	100 < dose ≤ 300 mg/day	4	-0.31	-0.50	-0.13	0.001*	0.0%	0.752	
	300 < dose ≤ 500 mg/day	7	-0.13	-0.23	-0.03	0.014*	11.3%	0.343	
	> 500 mg/day	1	0.00	-0.07	0.07	1.000	-	-	
	Health status								
	Obesity and Overweight	5	-0.08	-0.21	0.05	0.243	28.8%	0.229	0.281
	Insulin Resistance and Diabetes Mellitus	6	-0.17	-0.32	-0.02	0.030*	64.0%	0.016	
	Hypertension	-	-	-	-	-	-	-	
	Dyslipidemia	2	-0.06	-0.17	0.06	0.339	0.0%	0.399	
	Metabolic Syndrome	4	-1.22	-2.63	0.20	0.091	94.3%	0.000	
	Other	-	-	-	-	-	-	-	
	Formula								
	Purified anthocyanins	4	-0.22	-0.37	-0.08	0.003*	0.0%	0.401	0.253
	Anthocyanins-rich berries and their derivatives	6	-0.15	-0.30	0.00	0.047*	56.1%	0.044	
	Anthocyanin-rich fruit juices from other fruits	2	-0.10	-0.32	0.12	0.383	0.0%	0.375	
	Anthocyanin-rich foods and their derivatives	4	-1.12	-2.17	-0.07	0.037*	96.0%	0.000	
	Dosage frequency								
	Once a day	5	-0.10	-0.23	0.03	0.124	28.9%	0.229	0.313
	Twice a day	8	-0.08	-0.17	0.01	0.080	11.7%	0.339	
	Three times a day	-	-	-	-	-	-	-	
	Four times a day	1	-0.30	-0.57	-0.03	0.031*	-	-	
	N/A	2	-2.08	-5.42	1.26	0.222	98.2%	0.000	
	Physical activity levels								
	Maintain usual physical activity	4	-0.20	-0.42	0.02	0.080	0.0%	0.935	0.377
	Required to exercise a specific amount	2	-0.10	-0.32	0.12	0.383	0.0%	0.375	
	Avoid strenuous physical activity	1	-0.30	-0.57	-0.03	0.031*	-	-	
	N/A	9	-0.38	-0.63	-0.13	0.003*	91.3%	0.000	
	Baseline								
	> 5.6 mmol/L	10	-0.48	-0.78	-0.19	0.001*	90.3%	0.000	0.014
	≤ 5.6 mmol/L	6	-0.09	-0.19	0.00	0.055	0.0%	0.531	
Insulin	Overall effect	13	-0.02	-0.44	0.40	0.932	77.7%	0.000	
	Intervention duration								
	≤ 4 weeks	1	-0.82	-1.98	0.34	0.165	-	-	0.031
	4 < duration ≤ 8 weeks	4	0.60	-0.08	1.29	0.084	90.2%	0.000	
	8 < duration ≤ 12 weeks	6	-0.46	-1.30	0.37	0.279	64.7%	0.015	
	> 12 weeks	2	-1.44	-3.08	0.20	0.085	0.0%	0.415	
	Anthocyanins dosage								
	≤ 100 mg/day	4	-0.18	-0.78	0.43	0.569	5.9%	0.363	0.372
	100 < dose ≤ 300 mg/day	5	0.26	-0.58	1.10	0.539	90.2%	0.000	
	300 < dose ≤ 500 mg/day	3	-0.67	-1.54	0.21	0.134	0.0%	0.802	
	> 500 mg/day	1	0.05	-0.18	0.28	0.664	-	-	
	Health status								
	Obesity and Overweight	5	-0.16	-0.57	0.26	0.465	23.7%	0.263	0.616
	Insulin Resistance and Diabetes Mellitus	4	0.74	-1.01	2.49	0.405	88.0%	0.000	
	Hypertension	-	-	-	-	-	-	-	
	Dyslipidemia	-	-	-	-	-	-	-	
	Metabolic Syndrome	5	-0.07	-0.65	0.52	0.824	79.8%	0.001	
	Other	-	-	-	-	-	-	-	
	Formula								
	Purified anthocyanins	2	-0.49	-1.64	0.66	0.404	0.0%	0.643	0.309
	Anthocyanins-rich berries and their derivatives	7	0.23	-0.29	0.75	0.379	86.6%	0.000	
	Anthocyanin-rich fruit juices from other fruits	2	-1.20	-3.45	1.06	0.298	27.5%	0.240	
	Anthocyanin-rich foods and their derivatives	2	-0.35	-0.95	0.25	0.254	0.0%	0.357	
	Dosage frequency								
	Once a day	3	-0.93	-1.92	0.07	0.067	0.0%	0.473	0.149
	Twice a day	9	0.15	-0.33	0.64	0.540	82.6%	0.000	
	Three times a day	-	-	-	-	-	-	-	
	Four times a day	1	-0.21	-0.88	0.46	0.541	-	-	
	N/A	-	-	-	-	-	-	-	
	Physical activity levels								
	Maintain usual physical activity	5	0.17	-1.88	2.22	0.869	85.2%	0.000	0.680
	Required to exercise a specific amount	2	-1.20	-3.45	1.06	0.298	27.5%	0.240	
	Avoid strenuous physical activity	1	-1.50	-6.17	3.17	0.529	-	-	
	N/A	5	0.43	-1.69	2.54	0.692	85.2%	0.000	
HOMA-IR	Overall effect	8	-0.11	-0.51	0.28	0.573	87.4%	0.000	
	Intervention duration								
	≤ 4 weeks	-	-	-	-	-	-	-	0.000
	4 < duration ≤ 8 weeks	1	0.70	0.43	0.97	0.000*	-	-	
	8 < duration ≤ 12 weeks	6	-0.20	-0.60	0.20	0.332	78.3%	0.000	
	> 12 weeks	1	-0.46	-0.79	-0.13	0.006*	-	-	
	Anthocyanins dosage								
	≤ 100 mg/day	1	-0.20	-0.54	-0.54	0.248	-	-	0.501
	100 < dose ≤ 300 mg/day	3	0.10	-0.63	0.84	0.786	93.3%	0.000	
	300 < dose ≤ 500 mg/day	4	-0.33	-0.57	-0.09	0.007*	0.0%	0.675	
	> 500 mg/day	-	-	-	-	-	-	-	
	Health status								
	Obesity and Overweight	-	-	-	-	-	-	-	0.266
	Insulin Resistance and Diabetes Mellitus	4	-0.33	-0.57	-0.09	0.007*	0.0%	0.675	
	Hypertension	-	-	-	-	-	-	-	
	Dyslipidemia	-	-	-	-	-	-	-	
	Metabolic Syndrome	4	0.03	-0.56	0.62	0.920	92.2%	0.000	
	Other	-	-	-	-	-	-	-	
	Formula								
	Purified anthocyanins	4	-0.33	-0.57	-0.09	0.007*	0.0%	0.675	0.266
	Anthocyanins-rich berries and their derivatives	4	0.03	-0.56	0.62	0.920	92.2%	0.000	
	Anthocyanin-rich fruit juices from other fruits	-	-	-	-	-	-	-	
	Anthocyanin-rich foods and their derivatives	-	-	-	-	-	-	-	
	Dosage frequency								
	Once a day	-	-	-	-	-	-	-	0.598
	Twice a day	7	-0.09	-0.51	0.33	0.671	89.0%	0.000	
	Three times a day	-	-	-	-	-	-	-	
	Four times a day	-	-	-	-	-	-	-	
	N/A	1	-0.33	-1.11	0.45	0.409	-	-	
	Physical activity levels								
	Maintain usual physical activity	6	-0.09	-0.57	0.39	0.235	90.7%	0.000	0.807
	Required to exercise a specific amount	-	-	-	-	-	-	-	
	Avoid strenuous physical activity	-	-	-	-	-	-	-	
	N/A	2	-0.17	-0.57	0.24	0.413	0.0%	0.637	

a The pooled effects sizes and 95% CIs were calculated using the random-effects model.

b P value obtained from the subgroup analysis.

c * indicates statistically significant results in subgroup analysis (p < 0.05).

d ** indicates statistically significant results for the overall analysis (p < 0.05).

e Between-study heterogeneity was examined using the I-squared and Cochrane’s Q test.

Abbreviations: I—Intervention group, C—Control group; BMI—Body mass index; WC—Waist circumference; HDL-C—High-density lipoprotein cholesterol; LDL-C—Low-density lipoprotein cholesterol; TGs—Triglycerides; TC—Total cholesterol; FBG—Fasting blood glucose; HbA1c - Glycated hemoglobin; HOMA-IR—Homeostatic Model Assessment of Insulin Resistance.

### Dietary anthocyanins consumption and dyslipidemia

Based on 21 studies [31, 33, 52, 54, 58–64, 66–74, 77] (29 arms) involving 1995 participants, the results showed statistically significant heterogeneity for HDL-C outcomes (p = 0.000, I^2^ = 95.3%). Using a random-effects model, the intervention group showed a significantly greater increase in HDL-C levels compared to the control group (WMD: 0.05 mmol/L, 95% CI: 0.01 to 0.10, p = 0.026) (Fig 3D). Sensitivity analysis indicated that the significant effect of dietary anthocyanins on HDL-C became non-significant when the study by S. Kianbakht 2014 [68] was excluded (WMD: 0.04 mmol/L, 95% CI: -0.00 to 0.08, p > 0.05). Subgroup analysis revealed a significant increase in HDL-C with anthocyanin-rich fruit juices (WMD: 0.10 mmol/L, 95% CI: 0.01 to 0.19, p = 0.029), a dose of 100 < dose ≤ 300 mg/day (WMD: 0.09 mmol/L, 95% CI: 0.04 to 0.14, p = 0.000), and an intervention duration of 4 < duration ≤ 8 weeks (WMD: 0.08 mmol/L, 95% CI: 0.00 to 0.16, p = 0.040). Additionally, significant increases in HDL-C were observed in subgroups with baseline HDL-C ≤ 1.29 mmol/L (WMD: 0.07 mmol/L, 95% CI: 0.01 to 0.14, p = 0.036) and patients diagnosed with MetS (WMD: 0.08 mmol/L, 95% CI: 0.00 to 0.17, p = 0.042) (Table 2). Subgroup analyses based on dosage frequency and physical activity levels showed no significant changes.

Based on 21 studies [31, 33, 52, 54, 58–64, 66–74, 77] (29 arms) involving 1995 participants, the results indicated statistically significant heterogeneity for LDL-C outcomes (p = 0.000, I^2^ = 90.0%). Using a random-effects model, the intervention group showed a significantly greater reduction in LDL-C levels compared to the control group (WMD: -0.18 mmol/L, 95% CI: -0.28 to -0.08, p = 0.000) (Fig 3E). Sensitivity analysis confirmed that the overall effect on LDL-C remained stable upon exclusion of each study. Subgroup analysis revealed a significant reduction in LDL-C with anthocyanin-rich berries and their derivatives (WMD: -0.217 mmol/L, 95% CI: -0.341 to -0.093, p = 0.001), a dose > 500 mg/day (WMD: -0.184 mmol/L, 95% CI: -0.295 to -0.074, p = 0.001), an intervention duration of 4 < duration ≤ 8 weeks (WMD: -0.295 mmol/L, 95% CI: -0.457 to -0.133, p = 0.000), and where dosage frequency was not provided in the literature (WMD: -0.534 mmol/L, 95% CI: -0.774 to -0.294, p = 0.000). Additionally, significant reductions in LDL-C were observed in patients with insulin resistance and diabetes mellitus (WMD: -0.129 mmol/L, 95% CI: -0.246 to -0.011, p = 0.032), those with MetS (WMD: -0.484 mmol/L, 95% CI: -0.861 to -0.107, p = 0.012), and in studies where physical activity levels were not provided (WMD: -0.220 mmol/L, 95% CI: -0.357 to -0.084, p = 0.002) (Table 2).

15 studies [31, 54, 59–63, 66–70, 72, 73, 77], involving 21 trials and 1409 participants, revealed significant heterogeneity in TGs outcomes (p = 0.000, I^2^ = 87.9%). Using a random-effects model, the results indicated that dietary anthocyanin supplementation significantly improved TGs levels (WMD: -0.11 mmol/L, 95% CI: -0.20 to -0.02, p = 0.021) (Fig 3F). Sensitivity analysis showed that removing studies by Kim S Stote 2020b, Reza Mohtashami 2019, and S. Kianbakht 2014 [62, 67, 68] resulted in non-significant overall effects on TGs levels (WMD: -0.09 mmol/L, 95% CI: -0.19 to 0.00, p > 0.05; WMD: -0.08 mmol/L, 95% CI: -0.17 to 0.01, p > 0.05; and WMD: -0.09 mmol/L, 95% CI: -0.19 to 0.00, p > 0.05, respectively). Subgroup analysis revealed a significant reduction in TGs with a dose of 100 < dose ≤ 300 mg/day (WMD: -0.19 mmol/L, 95% CI: -0.31 to -0.07, p = 0.002), an intervention duration of 4 < duration ≤ 8 weeks (WMD: -0.23 mmol/L, 95% CI: -0.38 to -0.08, p = 0.002), and a dosage frequency of three times a day (WMD: -0.32 mmol/L, 95% CI: -0.45 to -0.19, p = 0.000). Additionally, significant reductions in TGs were observed in subgroups with baseline TGs > 1.7 mmol/L (WMD: -0.19 mmol/L, 95% CI: -0.34 to -0.04, p = 0.013), patients diagnosed with obesity and overweight (WMD: -0.13 mmol/L, 95% CI: -0.25 to 0.00, p = 0.046), and in studies where data on physical activity levels were not available (WMD: -0.14 mmol/L, 95% CI: -0.26 to -0.03, p = 0.014) (Table 2). Subgroup analyses based on the formula revealed no significant changes.

20 studies [31, 52–54, 58–62, 64, 66–74, 77], involving 28 trials and 1988 participants, reported statistically significant results for TC (p = 0.000, I^2^ = 95.2%). Therefore, a random-effects model was chosen. Combined results indicated that TC levels were significantly reduced in the intervention group compared to the control group (WMD: -0.34 mmol/L, 95% CI: -0.49 to -0.18, p = 0.000) (Fig 3G). Sensitivity analysis confirmed that the overall effect size remained consistent upon the exclusion of any single study. Subgroup analysis revealed a significant reduction in TC with anthocyanin-rich foods and their derivatives (WMD: -0.49 mmol/L, 95% CI: -0.93 to -0.06, p = 0.027), a dose of ≤ 100 mg/day (WMD: -0.41 mmol/L, 95% CI: -0.78 to -0.04, p = 0.032) and > 500 mg/day (WMD: -0.26 mmol/L, 95% CI: -0.36 to -0.15, p = 0.000), an intervention duration of 4 < duration ≤ 8 weeks (WMD: -0.36 mmol/L, 95% CI: -0.52 to -0.19, p = 0.000), and in studies where dosage frequency was not reported (WMD: -0.40 mmol/L, 95% CI: -0.53 to -0.26, p = 0.000). Additionally, significant reductions in TC were observed in patients with insulin resistance and diabetes mellitus (WMD: -0.15 mmol/L, 95% CI: -0.28 to -0.02, p = 0.026), and in studies where physical activity levels were not reported (WMD: -0.44 mmol/L, 95% CI: -0.63 to -0.26, p = 0.000) (Table 2).

### Dietary anthocyanins consumption and hypertension

Analysis of 21 studies [31, 39, 52, 56–58, 60–66, 70–77], involving 28 treatment groups and 1598 participants, revealed significant heterogeneity in SBP outcomes (p = 0.000, I^2^ = 64.2%). Therefore, a random-effects model was used. The results indicated no significant change in SBP levels in the intervention group compared to the control group (WMD: -0.12 mmHg, 95% CI: -1.06 to 0.82, p = 0.801) (Fig 3H). Sensitivity analysis showed that the overall effect on SBP was robust, with no single study significantly altering the results. Subgroup analysis revealed a significant increase in SBP with a dose of ≤ 100 mg/day (WMD: 2.73 mmHg, 95% CI: 1.40 to 4.06, p = 0.000) and in patients diagnosed with obesity and overweight (WMD: 2.27 mmHg, 95% CI: 0.51 to 4.02, p = 0.011). Additionally, significant decreases in SBP were observed with intervention durations of > 12 weeks (WMD: -3.96 mmHg, 95% CI: -7.60 to -0.32, p = 0.033) and in patients with dyslipidemia (WMD: -4.70 mmHg, 95% CI: -8.08 to -1.32, p = 0.006) (Table 2). However, subgroup analyses based on formula, dosage frequency, physical activity levels, and baseline SBP showed no significant changes.

For DBP, 21 studies [31, 39, 52, 56–58, 60–66, 70–77] involving 28 treatment groups and 1598 participants reported significant heterogeneity (p = 0.000, I^2^ = 72.1%). The random-effects model analysis showed no significant effect on DBP in the intervention group compared to the control group (WMD: 0.61 mmHg, 95% CI: -0.03 to 1.25, p = 0.061) (Fig 3I). Sensitivity analysis indicated that the removal of specific studies significantly altered the pooled effect size of dietary anthocyanins on DBP. Notably, excluding key trials such as those by Ah Jin Jung 2021 and April J Stull 2010 [60, 72] reversed the effect direction, highlighting their substantial impact on overall findings. Detailed results, including the impact of removing each of the 10 individual studies, are provided in S1 Fig in S2 File. Subgroup analysis revealed a significant increase in DBP with anthocyanin-rich berries and derivatives (WMD: 0.91 mmHg, 95% CI: 0.17 to 1.64, p = 0.015), doses > 500 mg/day (WMD: 0.91 mmHg, 95% CI: 0.14 to 1.68, p = 0.020), intervention duration ≤ 4 weeks (WMD: 1.72 mmHg, 95% CI: 1.02 to 2.42, p = 0.000), and dosage frequency either four times a day or not provided in the literature (WMD: 5.64 mmHg, 95% CI: 1.13 to 10.15, p = 0.014 and WMD: 1.49 mmHg, 95% CI: 1.24 to 1.74, p = 0.000, respectively). Additionally, significant increases in DBP were observed in patients with insulin resistance and diabetes mellitus (WMD: 1.27 mmHg, 95% CI: 0.55 to 2.00, p = 0.001), and in those who avoided strenuous physical activity (WMD: 5.64 mmHg, 95% CI: 1.13 to 10.15, p = 0.014) or had no reported physical activity levels (WMD: 0.99 mmHg, 95% CI: 0.28 to 1.70, p = 0.006) (Table 2). However, subgroup analyses based on baseline DBP revealed no significant changes.

### Dietary anthocyanins consumption and glucose-insulin regulation

For FBG, 13 studies [33, 52, 54–56, 60, 62, 63, 67, 69, 70, 72, 74] including 16 treatment groups with 1337 participants revealed significant heterogeneity (p = 0.000, I^2^ = 84.5%). A random-effects model showed that anthocyanin supplementation led to a significant reduction in FBG in the intervention group (WMD: -0.29 mmol/L, 95% CI: -0.46 to -0.12, p = 0.001) (Fig 3J). Sensitivity analysis indicated that no single study significantly influenced the overall FBG result. Subgroup analysis revealed significant reductions in FBG with purified anthocyanins, anthocyanin-rich berries, and anthocyanin-rich foods and derivatives (WMD: -0.22 mmol/L, 95% CI: -0.37 to -0.08, p = 0.003; WMD: -0.15 mmol/L, 95% CI: -0.30 to 0.00, p = 0.047; WMD: -1.12 mmol/L, 95% CI: -2.17 to -0.07, p = 0.037, respectively). Significant reductions were also observed with doses of ≤ 100 mg/day, 100 < dose ≤ 300 mg/day, and 300 < dose ≤ 500 mg/day (WMD: -0.95 mmol/L, 95% CI: -1.81 to -0.09, p = 0.031; WMD: -0.31 mmol/L, 95% CI: -0.50 to -0.13, p = 0.001; WMD: -0.13 mmol/L, 95% CI: -0.23 to -0.03, p = 0.014), and with intervention durations of 4 < duration ≤ 8 weeks and 8 < duration ≤ 12 weeks (WMD: -0.74 mmol/L, 95% CI: -1.27 to -0.20, p = 0.007; WMD: -0.18 mmol/L, 95% CI: -0.31 to -0.05, p = 0.006). Additionally, significant reductions in FBG were observed with a dosage frequency of four times a day (WMD: -0.30 mmol/L, 95% CI: -0.57 to -0.03, p = 0.031), among subgroups with baseline FBG > 5.6 mmol/L (WMD: -0.48 mmol/L, 95% CI: -0.78 to -0.19, p = 0.001), in patients with insulin resistance and diabetes mellitus (WMD: -0.17 mmol/L, 95% CI: -0.32 to -0.02, p = 0.030), and in those with physical activity levels classified as avoiding strenuous physical activity (WMD: -0.30 mmol/L, 95% CI: -0.57 to -0.03, p = 0.31) or not provided in the literature (WMD: -0.38 mmol/L, 95% CI: -0.63 to -0.13, p = 0.003) (Table 2).

For HbA1c, 4 studies [52, 54, 61, 67] including 5 treatment groups with 378 participants indicated significant heterogeneity (p = 0.000, I^2^ = 97.0%). A random-effects model showed that anthocyanin supplementation significantly affected HbA1c levels (WMD: -0.43%, 95% CI: -0.74 to -0.13, p = 0.005) (Fig 3K). Sensitivity analysis indicated that removing the study by Reza Mohtashami 2019 [67] changed the effect from significant improvement to non-significant (WMD: -0.11%, 95% CI: -0.23 to 0.01, p > 0.05). Due to the limited number of trials, no subgroup analysis was conducted.

Analysis from 10 studies [33, 52, 54, 60–63, 70, 72, 74] with 13 treatment groups and 847 participants revealed significant heterogeneity for insulin outcomes (p = 0.000, I^2^ = 77.7%). Using a random-effects model, no significant improvement in insulin levels was found in the intervention group (WMD: -0.02 mU/L, 95% CI: -0.44 to 0.40, p = 0.932) (Fig 3L). Sensitivity analysis showed that excluding any single study did not significantly affect the overall effect size on insulin levels. Subgroup analyses revealed no significant changes.

For HOMA-IR, 6 studies [52, 54–56, 61, 63] including 8 treatment groups with 558 participants showed significant heterogeneity (p = 0.000, I^2^ = 87.4%). The random-effects model indicated that anthocyanin intake had no significant effect on HOMA-IR scores (WMD: -0.11, 95% CI: -0.51 to 0.28, p = 0.573) (Fig 3M). Sensitivity analysis confirmed that excluding any single study did not significantly alter the statistical results for this measure. Subgroup analysis revealed a significant reduction in HOMA-IR with purified anthocyanins (WMD: -0.33, 95% CI: -0.57 to -0.09, p = 0.007), at doses of 300 < dose ≤ 500 mg/day (WMD: -0.33, 95% CI: -0.57 to -0.09, p = 0.007), in patients with insulin resistance and diabetes mellitus (WMD: -0.33, 95% CI: -0.57 to -0.09, p = 0.007), and for intervention durations > 12 weeks (WMD: -0.46, 95% CI: -0.79 to -0.13, p = 0.006). A significant increase in HOMA-IR was observed for intervention durations of 4 < duration ≤ 8 weeks (WMD: 0.70, 95% CI: 0.43 to 0.97, p = 0.000) (Table 2). Subgroup analyses based on dosage frequency and physical activity levels revealed no significant changes.

### Publication bias

**Fig 4:**
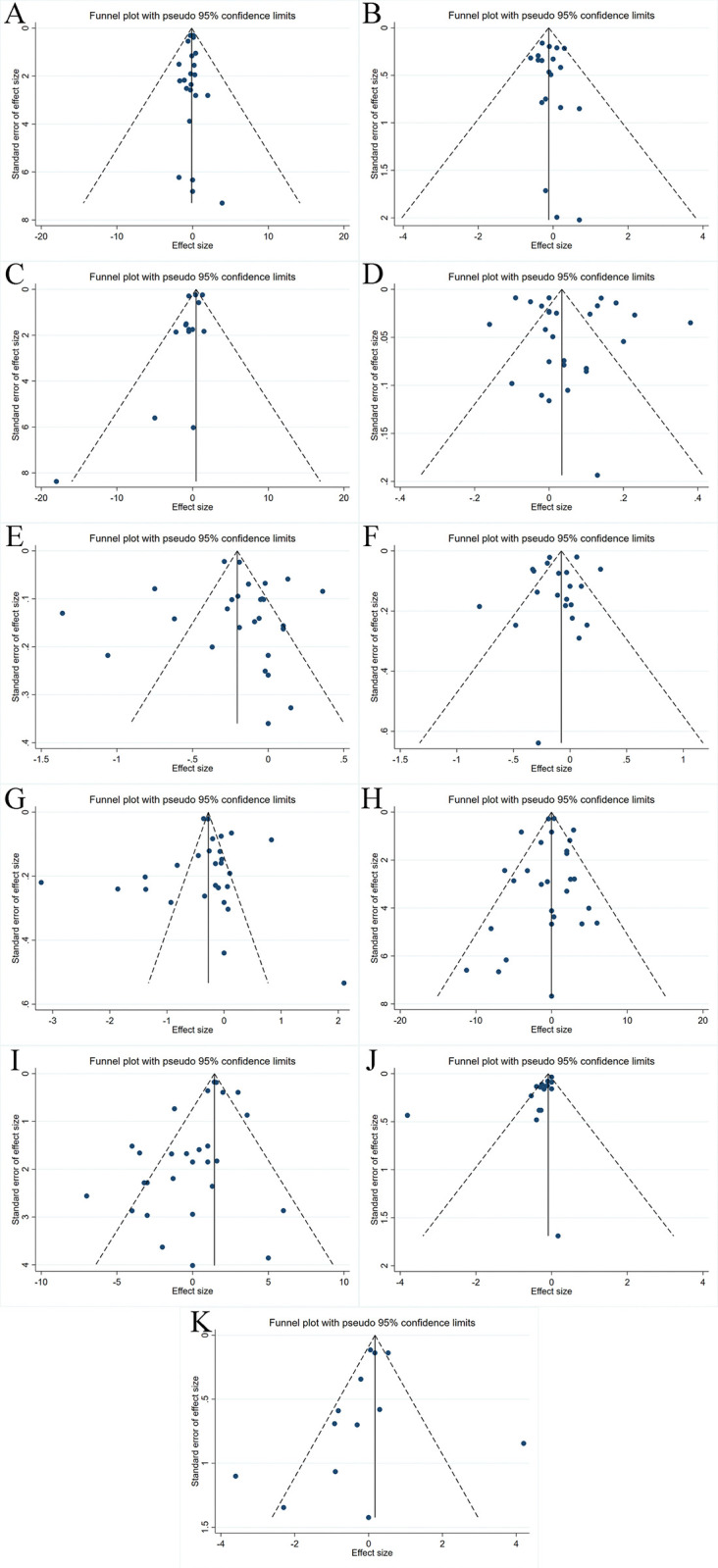
Funnel plot. Funnel plots illustrating the effect of dietary anthocyanin consumption on (A) Weight (kg), (B) BMI (kg/m^2^), (C) WC (cm), (D) HDL-C (mmol/L), (E) LDL-C (mmol/L), (F) TGs (mmol/L), (G) TC (mmol/L), (H) SBP (mmHg), (I) DBP (mmHg), (J) FBG (mmol/L), and (K) Insulin (mU/L).

Except for DBP and FBG, visual inspection of funnel plots for variables such as WC, HDL-C, LDL-C, TGs, TC, SBP, and insulin showed slight asymmetry. However, Egger’s test indicated no significant evidence of bias in the meta-analyses for dietary anthocyanin supplementation effects on weight (p = 0.682) (Fig 4A), BMI (p = 0.682) (Fig 4B), WC (p = 0.140) (Fig 4C), HDL-C (p = 0.530) (Fig 4D), LDL-C (p = 0.555) (Fig 4E), TGs (p = 0.571) (Fig 4F), TC (p = 0.958) (Fig 4G), SBP (p = 0.665) (Fig 4H), and insulin (p = 0.435) (Fig 4K). In contrast, both funnel plots and Egger’s test revealed significant publication bias for DBP (p = 0.014) (Fig 4I) and FBG (p = 0.017) (Fig 4J). Therefore, the trim-and-fill method was used to adjust the pooled results for DBP and FBG. After adjustment, the effect sizes for DBP and FBG remained (WMD: 0.61 mmHg, 95% CI: -0.03 to 1.25, p = 0.061) and (WMD: -0.29 mmol/L, 95% CI: -0.46 to -0.12, p = 0.001), respectively, indicating no significant publication bias in the original data S2 and S3 Figs in S2 File.

## Discussion

### Major discoveries

This systematic review and meta-analysis included 29 randomized controlled trials involving 2006 participants to assess the effects of dietary anthocyanins on obesity indices, lipid profile, blood pressure, and glycemic control in patients with risk factors for metabolic syndrome. This is the first systematic review and meta-analysis to examine the impact of dietary anthocyanins on metabolic syndrome risk factors. The results demonstrated that anthocyanin supplementation significantly reduced LDL-C, TGs, TC, FBG, and HbA1c levels, and increased HDL-C levels, but did not show significant effects on weight, BMI, WC, SBP, DBP, insulin, and HOMA-IR levels. Sensitivity analyses indicated that the originally non-significant effects on BMI and DBP reversed to significant decreases and increases, respectively, upon the exclusion of a single study. However, the effects on HDL-C, TGs, and HbA1c changed from significant to non-significant when individual studies were sequentially excluded.

### Effects of dietary anthocyanins on obesity indices

This meta-analysis investigated the effects of dietary anthocyanin supplementation on obesity indices. The current meta-analysis did not find that anthocyanin supplementation significantly reduced weight, BMI, and WC.

Tirang et al. (2023) conducted a systematic review and meta-analysis to evaluate the impact of various forms of anthocyanin supplementation on cardiometabolic risk factors in humans, finding significant effects on BMI and body fat mass but not on WC [29]. Similarly, two other studies reported comparable findings. Wilken et al. (2022) performed a systematic review and meta-analysis on anthocyanin-rich berries in patients with metabolic syndrome, showing a significant decrease in BMI post-intervention [30]. Park et al. (2021) also found that anthocyanin supplementation significantly impacted BMI but not weight or WC [78]. Despite the beneficial effects highlighted in these systematic reviews and meta-analyses, some studies reported contradictory results. For example, Xu et al. observed no impact on BMI from purified anthocyanins or anthocyanin-rich berries [79]. These findings have been reiterated in recent meta-analyses, with other anthropometric measurements such as waist-hip ratio, percentage of fat mass, hip circumference, and fat-free mass showing no significant changes [80, 81].

Our systematic review results align with those of Yarhosseini et al. [80], but our meta-analysis exhibits lower heterogeneity, likely due to stricter inclusion and exclusion criteria. Although we did not observe significant effects of dietary anthocyanins on obesity indices, subgroup analysis revealed a significant reduction in WC at intervention doses ≤100 mg/day. This effective dose range aligns with the Chinese Nutrition Society’s recommended daily dietary reference intake of 50 mg for anthocyanins [82]. Additionally, literature indicates that anthocyanin concentrations of 10–50 μg/mL optimally exhibit anti-inflammatory effects in vitro, while higher doses (10 mM) may be toxic and reduce cell viability [83]. This might explain the lack of significant effects at doses >100 mg/day.

Furthermore, numerous studies have reported the mechanisms through which anthocyanins or anthocyanin-rich berries improve obesity. Anthocyanins, a major subclass of flavonoids, possess strong antioxidant properties due to their phenolic hydroxyl groups [84, 85]. Natural substances with antioxidant properties can promote browning of adipose tissue and reduce obesity by regulating reactive oxygen species (ROS), which are considered regulators of browning [86]. The conversion of white adipocytes to beige adipocytes has been observed, leading to an increase in beige fat cells, which burn nutrients and thus reduce obesity [87].

Additionally, as dietary polyphenols, anthocyanins have been shown to influence the growth of colonic bacteria [88]. Anthocyanins and their metabolites produced in the gut can act as antimicrobials, inhibiting the growth of harmful bacteria and promoting beneficial bacteria such as Lactobacillus spp., Bifidobacterium spp., and Akkermansia muciniphila spp [89, 90]. Studies have demonstrated that the probiotic Lactiplantibacillus plantarum can safely and effectively reduce body fat [91].

### Effects of dietary anthocyanins on lipid profile

This meta-analysis examined the effects of dietary anthocyanin supplementation on lipid profiles. The study found that supplementation with dietary anthocyanins significantly reduced LDL-C, TGs, and TC levels and significantly increased HDL-C levels.

Previous meta-analyses evaluated the effects of tart cherry juice and cranberry, which contain anthocyanins as their primary bioactive compounds, on metabolic health markers. These studies did not show significant effects on HDL-C, LDL-C, TGs, and TC levels [37, 81]. Interestingly, cranberry interventions significantly reduced the TC/HDL-C ratio, a more effective predictor of CVD incidence [37, 92]. Recent meta-analyses have assessed the impact of anthocyanin supplementation on lipid levels in participants with T2DM [28], MetS [22, 30], and unhealthy participants (overweight/obesity, prediabetes, and T2DM) [93], as well as those without specific health conditions [18, 29]. The results indicated varying degrees of lipid profile improvement, but consensus on the effects on HDL-C and TC levels was not achieved.

According to this study, anthocyanin supplements may be an effective therapeutic approach for increasing HDL-C and reducing LDL-C, TGs, and TC levels. The observed effects on lipid profiles in this study align only with those reported by Neyestani et al. [29]. The discrepancies with other studies may be attributed to differences in the sources of anthocyanins and their varying efficacy in regulating lipid levels among different health populations. Our subgroup analysis provides support for these potential reasons. Different dietary anthocyanin interventions, such as anthocyanin-rich berries and their derivatives, anthocyanin-rich fruit juices from other fruits, and anthocyanin-rich foods and their derivatives, showed significant effects on LDL-C, HDL-C, and TC. Dietary anthocyanin supplementation significantly improved lipid profiles in individuals with MetS, insulin resistance and diabetes mellitus, and obesity and overweight, as well as in those with risk factors for metabolic syndrome, such as baseline HDL-C ≤ 1.29 mmol/L and baseline TGs > 1.7 mmol/L.

Additionally, our results showed that dietary anthocyanin supplementation significantly improved HDL-C, LDL-C, TGs, and TC levels only when the intervention duration was 4 < duration ≤ 8 weeks. This may be due to compensatory mechanisms that mitigate the effects of prolonged anthocyanin activation. Furthermore, subgroup analysis revealed that daily anthocyanin supplementation at 100 < dose ≤ 300 mg/day significantly increased HDL-C and decreased TGs levels, while doses > 500 mg/day significantly decreased LDL-C levels. The observed differences in the effects of varying doses may be attributed to the different effective concentrations of anthocyanins required to modulate distinct mechanisms that improve the lipid profile. However, TC significantly decreased at daily doses ≤ 100 mg/day and > 500 mg/day, while no changes were observed at moderate doses (100 < dose ≤ 300 mg/day), possibly due to a significant increase in HDL-C levels within this dose range.

The mechanisms underlying these lipid profile improvements are still under investigation. Anthocyanins exert a comprehensive range of effects on lipid metabolism, contributing to their lipid-lowering effects. This complex mechanism primarily involves the regulation of key enzymes and receptors in hepatocytes, highlighting their potential as therapeutic agents in managing hyperlipidemia.

Firstly, some studies suggest that the core of anthocyanins’ lipid-lowering effects is the activation of AMP-activated protein kinase (AMPK), a crucial regulator of cellular energy homeostasis. Anthocyanins lower the expression of fatty acid synthase and HMG-CoA reductase, which are critical for lipid synthesis, through AMPK activation. Additionally, anthocyanins inhibit the activity of sterol regulatory element-binding proteins (SREBPs), particularly SREBP-1, thus reducing the transcription of lipogenic enzymes and subsequent lipid synthesis [94]. Furthermore, anthocyanins regulate CCAAT/enhancer-binding proteins (C/EBPs), preventing the differentiation and maturation of adipocytes by inhibiting C/EBPα, thereby reducing lipid accumulation [95]. Moreover, anthocyanins enhance the expression and function of low-density lipoprotein receptors (LDLR) in hepatocytes, promoting the clearance of LDL cholesterol from the bloodstream and reducing lipid levels [94]. This effect is further enhanced through the regulation of liver X receptor alpha (LXR-α) activity, downregulating LXR-α to inhibit new lipid synthesis and promoting cholesterol excretion and transport, leading to HDL formation and increased hepatic uptake of LDL [95, 96]. Additionally, anthocyanins modulate peroxisome proliferator-activated receptors (PPARs), particularly PPAR-α and PPAR-γ [97]. PPAR-α activation promotes lipolysis and fatty acid oxidation in the liver and adipose tissue, while PPAR-γ plays a critical role in lipid storage and insulin sensitivity [95]. Anthocyanins also influence lipid metabolism by altering adiponectin levels, increasing adiponectin and reducing leptin and anti-leptin levels, further supporting lipid homeostasis and cardiovascular health [97].

### Effects of dietary anthocyanins on blood pressure

This meta-analysis shows that, when considering the pooled effect size, anthocyanin supplementation overall did not affect SBP or DBP. However, it is noteworthy that the effect size for DBP showed a significant increase after adjustment using the trim-and-fill method. Ting et al. (2023) also reported that anthocyanin supplementation had no significant effect on SBP and DBP in their meta-analysis [28]. These findings are consistent with previous systematic reviews and meta-analyses [22, 30, 79, 81]. Furthermore, subgroup analyses in these studies did not reveal any significant differences based on the stratified factors of the research topics.

However, Igwe et al. (2019) found in a prospective cohort study of 12,153 residents in non-remote areas of Australia that anthocyanin intake was significantly inversely correlated with SBP and DBP in adults aged 50 years and above [98]. Similarly, Sedigheh et al. (2013) demonstrated in a clinical study that anthocyanin-rich pomegranate juice had potential cardioprotective effects for hypertensive patients, particularly by reducing SBP, DBP, and serum VCAM-1 levels [99].

The mechanisms by which anthocyanins exert their effects involve multiple pathways. Some studies suggest that anthocyanins delay cellular aging by inhibiting p53 expression and ROS production, thereby preventing eNOS uncoupling and promoting NO production and bioavailability [100]. Subsequently, endothelium-derived NO diffuses to vascular smooth muscle cells (VSMCs), where it activates the cGMP-protein kinase G (PKG) axis, stimulating Ca^2+^-activated potassium channels. This process induces membrane hyperpolarization and inhibits extracellular Ca^2+^ influx and/or Ca^2+^ release from the endoplasmic reticulum, leading to vasodilation [101].

Additionally, clinical studies have shown that anthocyanin supplementation can significantly increase serum adiponectin concentrations in patients with type 2 diabetes [52]. Evidence indicates that adiponectin plays a critical role in blood pressure regulation by preventing hypertension through endothelium-dependent mechanisms and by reversing salt-induced hypertension [102].

Our subgroup analysis found that dietary anthocyanin interventions significantly reduced SBP in dyslipidemic subjects and when the intervention duration exceeded 12 weeks. Conversely, SBP increased significantly when the dosage was ≤100 mg/day and the subjects were obese or overweight. Moreover, DBP increased significantly when the intervention duration was ≤4 weeks, the dosage exceeded 500 mg/day, the subjects had insulin resistance or diabetes mellitus, or the intervention involved anthocyanin-rich berries and their derivatives.

The elevated blood pressure findings primarily originated from four studies [60–62, 74], which, while showing no significant differences in their original data, indicated a significant increase in BP upon subgroup analysis in our study. Indeed, other meta-analyses have also reported non-significant pooled results for SBP and DBP, though with a trend towards an increase. However, due to the limited number of included studies, these analyses could not perform subgroup analysis to further investigate effect modifiers and sources of heterogeneity [22]. The potential hypertensive effects of dietary anthocyanins under specific conditions warrant caution and highlight the need for further research into the mechanisms by which dietary anthocyanins regulate blood pressure.

### Effects of dietary anthocyanins on glucose-insulin regulation

This meta-analysis indicates that anthocyanins have a significant overall effect on FBG and HbA1c levels but do not significantly impact insulin and HOMA-IR. Wilken et al. found that anthocyanin-rich berries had no significant effect on glycemic indices in patients with metabolic syndrome [30]. Similar results were reported by Araya-Quintanilla et al. [22]. Recent studies evaluating the effects of anthocyanin supplementation on blood glucose regulation and insulin sensitivity show significant improvements in glycemic control indices such as FBG and HbA1c, but no significant impact on insulin sensitivity markers like insulin and HOMA-IR [28, 29, 81, 93]. Interestingly, a recent study revealed that cranberry supplementation significantly reduced HOMA-IR without affecting HbA1c and insulin levels [37]. Mao et al. found that anthocyanin intervention significantly lowered 2-hour postload glucose (2h-PG), an important marker for assessing diabetes risk and complications [37, 103]. Overall, there is considerable inconsistency in the findings.

The overall results of this study align with four recent meta-analyses; however, it is noteworthy that dietary anthocyanin interventions significantly improved FBG and HOMA-IR in subjects with insulin resistance and diabetes mellitus, and in those with metabolic syndrome-related factors (FBG > 5.6 mmol/L). Purified anthocyanins were particularly effective in reducing FBG and HOMA-IR levels, showing stronger improvements compared to other forms of anthocyanins. Anthocyanin-rich berries, containing vitamins such as vitamin C and vitamin E and other bioactive compounds like carotenoids, may synergistically improve glycemic control [104]. The bioavailability of anthocyanins is as low as 1%, suggesting that the observed beneficial effects of anthocyanin-rich berries may be attributed to other bioactive components within the berries rather than anthocyanins alone [105]. However, our findings suggest that anthocyanins are likely the primary contributors to the improvement in glycemic control and insulin sensitivity.

Several studies indicate that anthocyanins effectively inhibit α-glucosidase activity, an enzyme responsible for carbohydrate breakdown in the intestine, thus slowing glucose absorption and reducing postprandial blood glucose fluctuations [106, 107]. There is also evidence that anthocyanins can affect glucose absorption in the intestine through active sodium-dependent transport mechanisms by inhibiting sodium-glucose cotransporter 1 (SGLT1) and downregulating glucose transporter 2 (GLUT2) in human intestinal Caco-2 cells, thereby inhibiting both sodium-dependent and non-sodium-dependent glucose transport [106, 108]. Furthermore, anthocyanins can increase cholesterol precipitation in Caco-2 cells, competitively inhibiting cholesterol uptake and thereby affecting blood lipid levels [108].

In vitro studies have shown that anthocyanins activate HepG2 cells by upregulating FOXO1 and PGC-1α expression, reducing phosphoenolpyruvate carboxykinase (PEPCK) and glucose-6-phosphatase (G6Pase) activity, thus decreasing gluconeogenesis and countering insulin resistance [108–110]. Anthocyanins also activate PPARγ in adipocytes, inducing GLUT4 translocation to enhance glucose uptake and glycogen synthesis [108–110]. PPARγ regulates adiponectin, and anthocyanins have been shown to increase serum adiponectin levels, a major player in regulating glucose and lipid metabolism in skeletal muscle and liver as an insulin sensitizer [109–112]. These mechanisms collectively lead to reduced FBG levels.

Recent studies found that Aronia melanocarpa anthocyanin extracts enhance insulin sensitivity and liver glycogen synthesis while reducing inflammation, oxidative stress, and liver damage by inhibiting the IKKβ/NF-κB p65, JAK2/Stat3/5B, and insulin/Stat5B signaling pathways [113]. Additionally, Chinese bayberry extract rich in anthocyanins was shown to protect β-cells from oxidative damage by activating heme oxygenase-1, altering the PI3K/Akt and ERK1/2 pathways, and inhibiting β-cell apoptosis [114].

### Strengths and limitations

This meta-analysis has several strengths and limitations. First, it is the first meta-analysis of randomized controlled trials examining the effects of dietary anthocyanin supplementation on metabolic syndrome components in patients at risk for metabolic syndrome. Previous meta-analyses have investigated the impact of anthocyanin-rich foods on cardiometabolic factors in MetS patients and the effect of anthocyanin-rich berries on MetS risk, but these studies were limited to participants already diagnosed with MetS. Our meta-analysis included patients with MetS risk factors, thus expanding the sample size and incorporating five recently published trials. This focus on the prevention and treatment of MetS enhances the credibility of our findings. Additionally, the combined results of this study indicate no publication bias, further strengthening our findings. We also performed a comprehensive subgroup analysis with seven stratifications, including the innovative inclusion of dosage frequency and physical activity levels, to thoroughly explore heterogeneity and provide robust evidence for precise nutritional intake recommendations. However, this study also has some limitations. First, there was high heterogeneity, and we did not conduct meta-regression or non-linear dose-response analysis to assess the impact of anthocyanin dosage and intervention duration on the outcomes. Second, subgroup analysis for weight and BMI was not performed, limiting the exploration of effect modification by these variables. Third, the sensitivity analysis indicated instability in the pooled results for BMI, HDL-C, TGs, DBP, and HbA1c.

## Conclusions

This meta-analysis demonstrates that dietary anthocyanin supplementation significantly improves serum levels of HDL-C, LDL-C, TGs, TC, FBG, and HbA1c. It also significantly ameliorates three MetS-related risk factors: low HDL cholesterol, high triglycerides, and hyperglycemia. These findings suggest that dietary anthocyanins may offer promising adjunctive benefits for preventing or treating MetS. However, it is important to note that dietary anthocyanin interventions may raise SBP and DBP depending on intervention dose, duration, participant health status, and formulation. Clinicians should thoroughly consider these potential hypertensive effects when recommending dietary anthocyanins to patients with MetS risk factors. Further well-designed RCTs and mechanistic studies are recommended to clarify the effects of dietary anthocyanins on metabolic syndrome risk factors, particularly regarding different intervention doses, durations, and physical activity levels.

## Supporting information

S1 Checklist PRISMA 2020 checklist. (DOCX)

S1 File The methods of subgroup analysis and sensitivity analysis in this study. (DOCX)

S2 File (ZIP)

S3 File Original study data. (RAR)

S1 Table Common definitions and criteria for metabolic syndrome. (XLSX)

S2 Table Summary of studies identified in the literature search, including studies excluded from the analysis and reasons for exclusion. (XLSX)

S3 Table Data extracted from included studies for the systematic review and meta-analysis. (XLSX)

S4 Table Specifications and outcomes of clinical trial studies included in the meta-analysis. (XLSX)

S5 Table Risk of bias assessment for eligible randomized controlled trials. (XLSX)

S6 Table Results of the effect of dietary anthocyanins on health outcomes, including effect size and heterogeneity analysis. (XLSX)
