# Supplementary material for: Effect of dietary anthocyanins on the risk factors related to metabolic syndrome: A systematic review and meta-analysis
Source: PLoS One. 2025 Feb 10;20(2):e0315504. doi: 10.1371/journal.pone.0315504 (PMC11809928; doi:10.1371/journal.pone.0315504)
Supplement: S1 File — (DOCX) [file pone.0315504.s002.docx]

**Dear Reviewer,**

Thank you for reviewing our manuscript and for your valuable comments. In response to your query regarding the methods of our subgroup analyses and sensitivity analyses, we provide the following detailed explanation.

**Subgroup Analysis Methods:**

1. Objective:

To explore potential sources of heterogeneity among the studies, we conducted subgroup analyses on the following parameters: waist circumference (WC), high-density lipoprotein cholesterol (HDL-C), low-density lipoprotein cholesterol (LDL-C), triglycerides (TGs), total cholesterol (TC), systolic blood pressure (SBP), diastolic blood pressure (DBP), fasting blood glucose (FBG), glycated hemoglobin (HbA1c), insulin, and the homeostasis model assessment of insulin resistance (HOMA-IR).

2. Subgroup Classification:

We performed subgroup analyses based on the following factors:

- Baseline Levels: According to the diagnostic criteria of metabolic syndrome, participants were divided into high and low baseline level subgroups. For example:

- HDL-C: High baseline level (> 1.29 mmol/L) and low baseline level (≤ 1.29 mmol/L).

- Other indicators: Classified according to corresponding cutoff values.

- Intervention Dosage: ≤ 100 mg/day; 100 < dose ≤ 300 mg/day; 300 < dose ≤ 500 mg/day; > 500 mg/day.

- Administration Frequency: Once a day; Twice a day; Three times a day; Four times a day; Not provided.

- Duration of Intervention: ≤ 4 weeks; 4 < duration ≤ 8 weeks; 8 < duration ≤ 12 weeks; > 12 weeks.

- Intervention Formulation: Purified anthocyanins; Anthocyanin-rich berries and their derivatives; Anthocyanin-rich fruit juices from other fruits; Anthocyanin-rich foods and their derivatives.

- Physical Activity Levels: Maintain usual physical activity; Required to exercise a specific amount; Avoid strenuous physical activity; Not provided.

- Health Status: Obesity and Overweight; Insulin Resistance and Diabetes Mellitus; Hypertension; Dyslipidemia; Metabolic Syndrome; Other.

3. Statistical Methods:

Within each subgroup, we performed meta-analyses using the DerSimonian-Laird random-effects model to calculate the weighted mean differences (WMDs) and their 95% confidence intervals (CIs).

4. Heterogeneity Assessment:

We assessed heterogeneity within each subgroup using Cochran's Q test and the I² statistic. Additionally, we employed the Q test to compare differences between subgroups, evaluating the impact of specific factors on the overall effect.

**Sensitivity Analysis Methods:**

1. Objective:

To verify the robustness of our findings and ensure that no single study unduly influenced the combined effect size, we conducted sensitivity analyses.

2. Methods:

- One-by-One Exclusion Method: We sequentially excluded each included study and recalculated the combined effect size (WMD) and its 95% CI to observe whether the exclusion of that study led to significant changes in the combined effect size and confidence interval. Throughout the sensitivity analyses, we employed the DerSimonian-Laird random-effects model, as this model effectively handles heterogeneity among studies. Considering the potential variability across studies, the random-effects model allows us to incorporate heterogeneity into the analysis, yielding more robust effect estimates.

3. Evaluation Criteria:

If the results of the sensitivity analyses are consistent with the main analysis, and the combined effect size and confidence intervals do not change significantly, we consider the results to be robust.
