# Supplementary figures and images for: Effect of dietary anthocyanins on the risk factors related to metabolic syndrome: A systematic review and meta-analysis
Source: PLoS One. 2025 Feb 10;20(2):e0315504. doi: 10.1371/journal.pone.0315504 (PMC11809928; doi:10.1371/journal.pone.0315504)

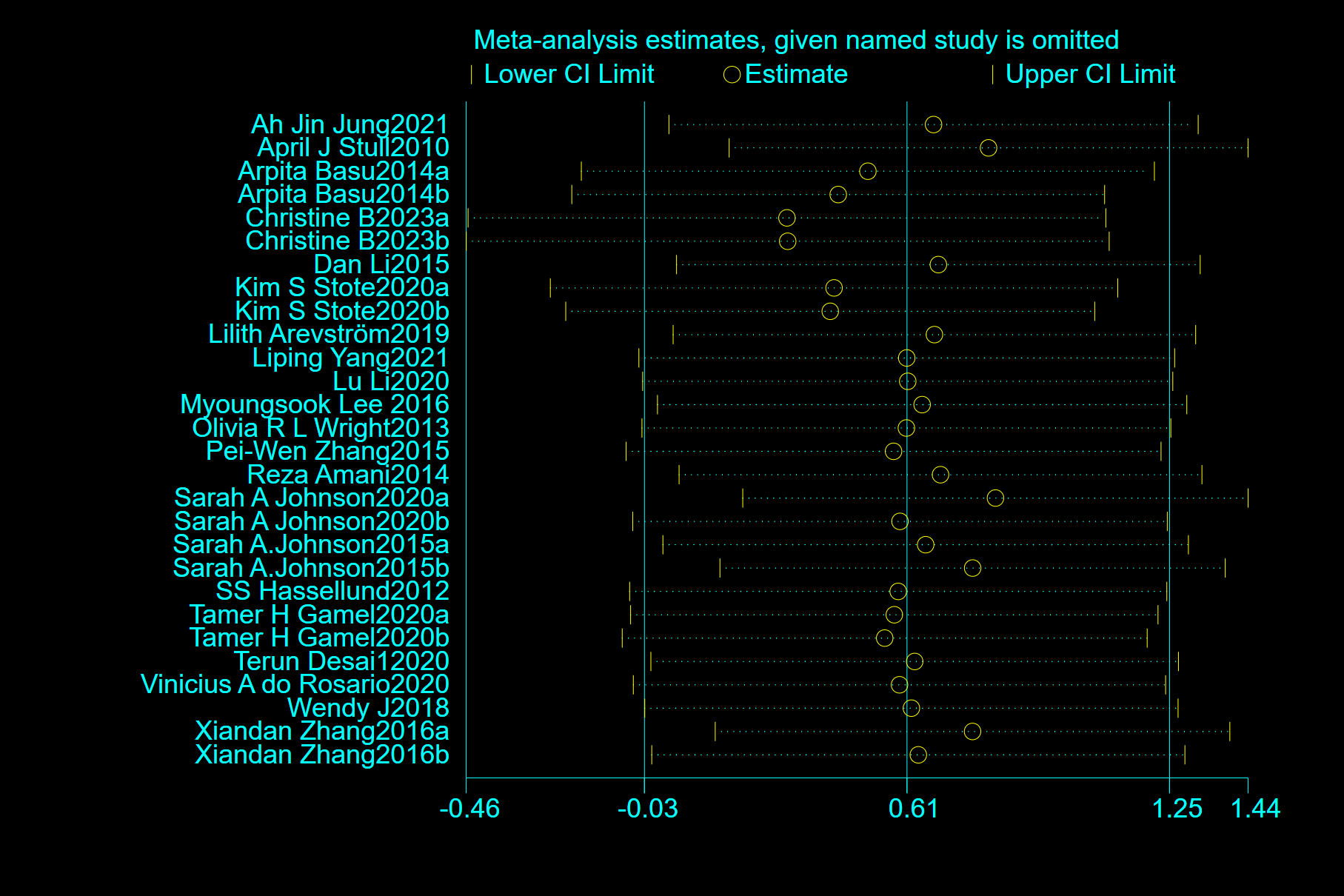

Supplement: S2 File — (ZIP) [file pone.0315504.s003.zip › S2 File/S1 Fig/S1 Fig_Sensitivity Analysis Plot for DBP.tif]

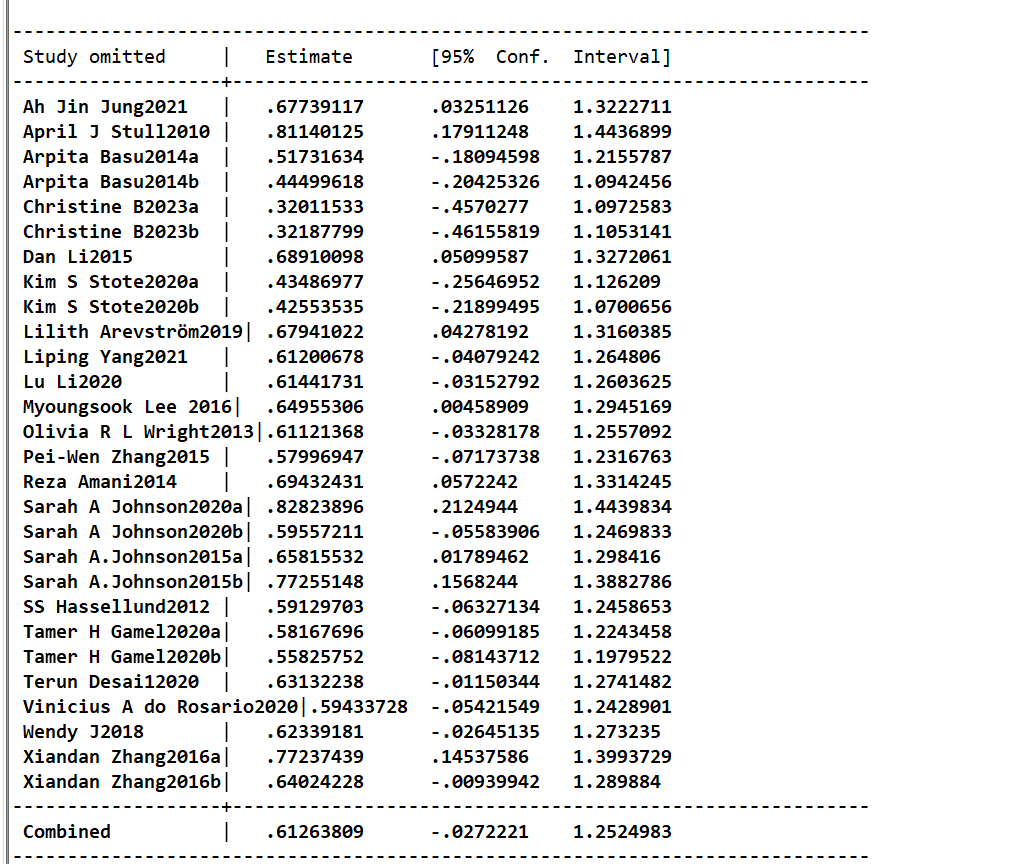

Supplement: S2 File — (ZIP) [file pone.0315504.s003.zip › S2 File/S1 Fig/Sensitivity Analysis Data for DBP.jpg]

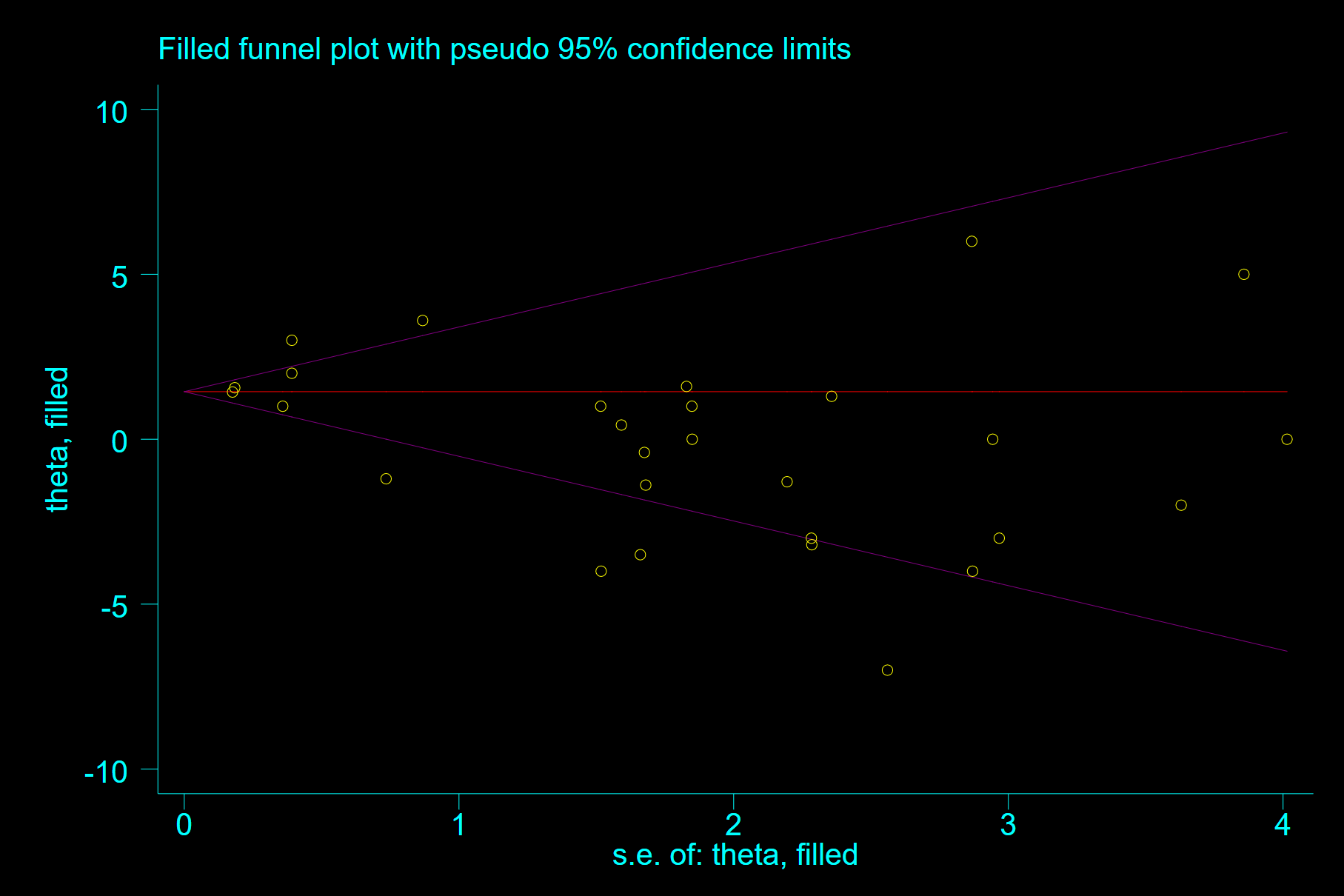

Supplement: S2 File — (ZIP) [file pone.0315504.s003.zip › S2 File/S2 Fig/S2 Fig_Trim-and-Fill Plot for DBP.tif]

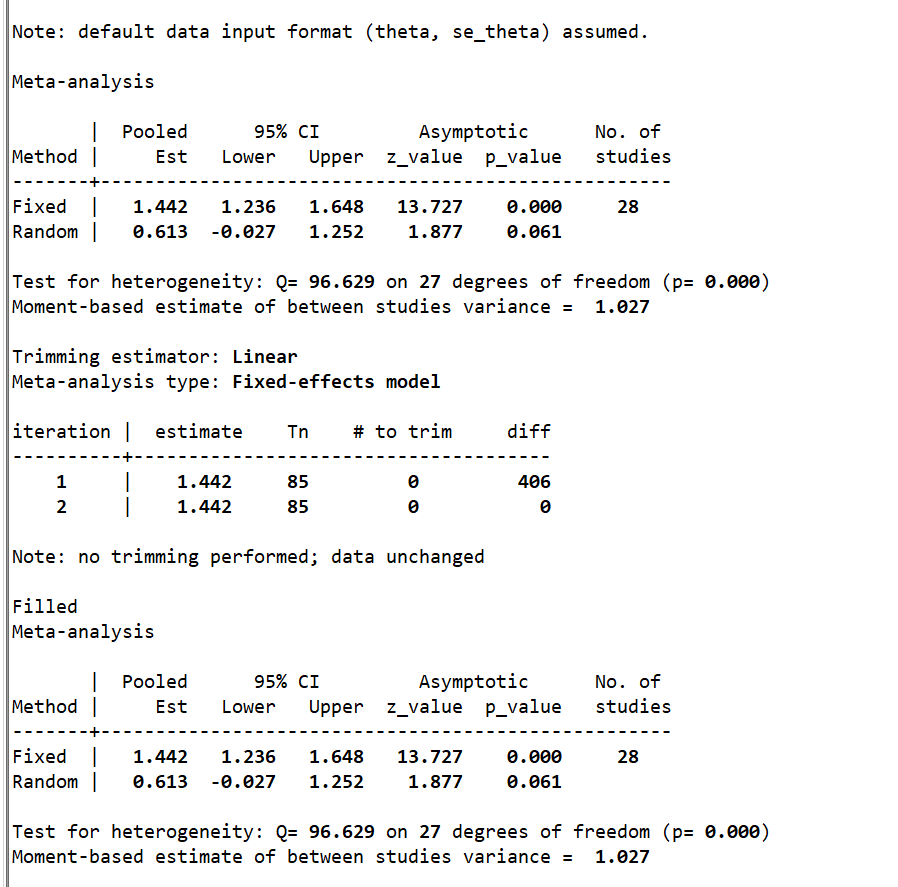

Supplement: S2 File — (ZIP) [file pone.0315504.s003.zip › S2 File/S2 Fig/Trim-and-Fill Data for DBP.jpg]

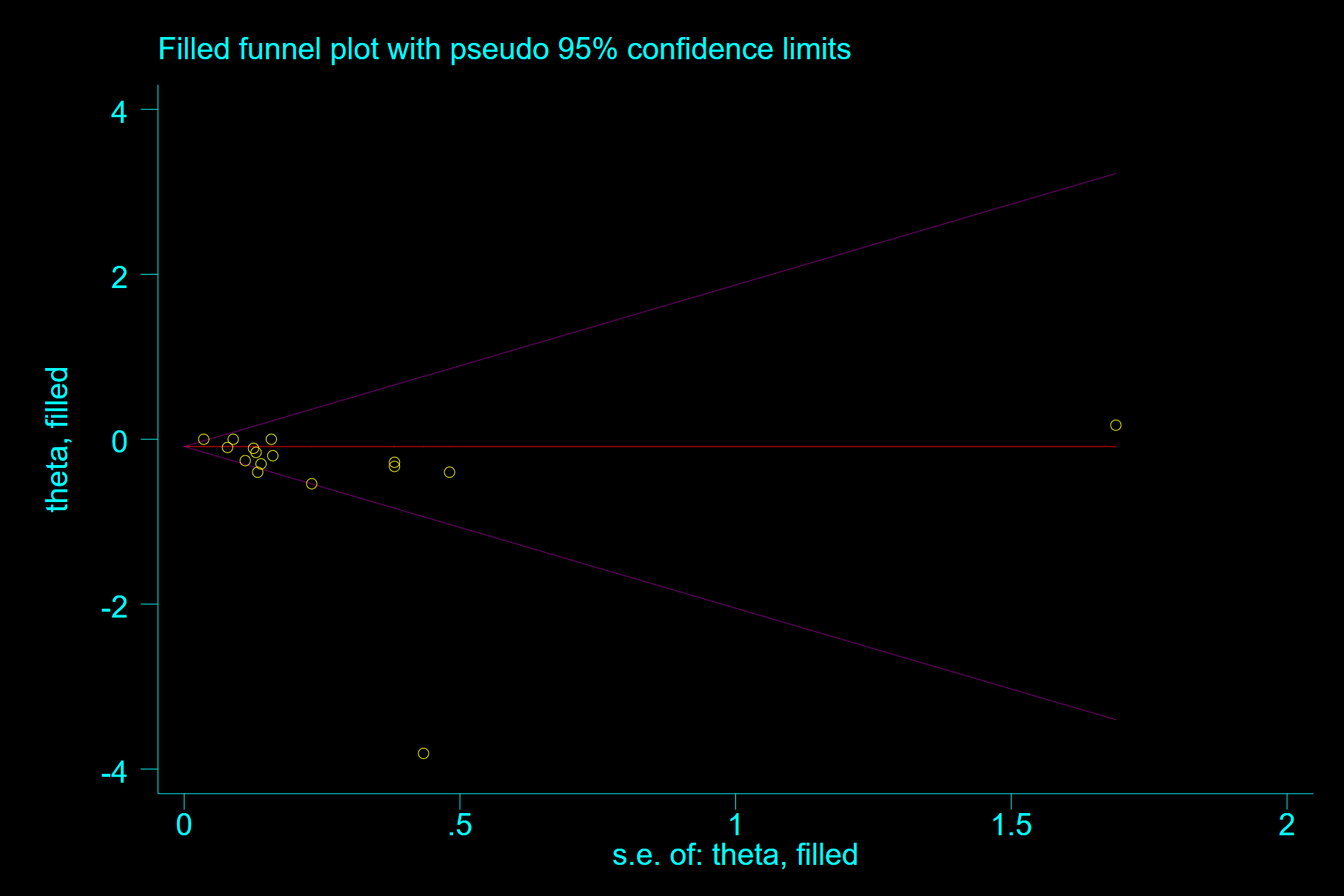

Supplement: S2 File — (ZIP) [file pone.0315504.s003.zip › S2 File/S3 Fig/S3 Fig_Trim-and-Fill Plot for FBG.tif]

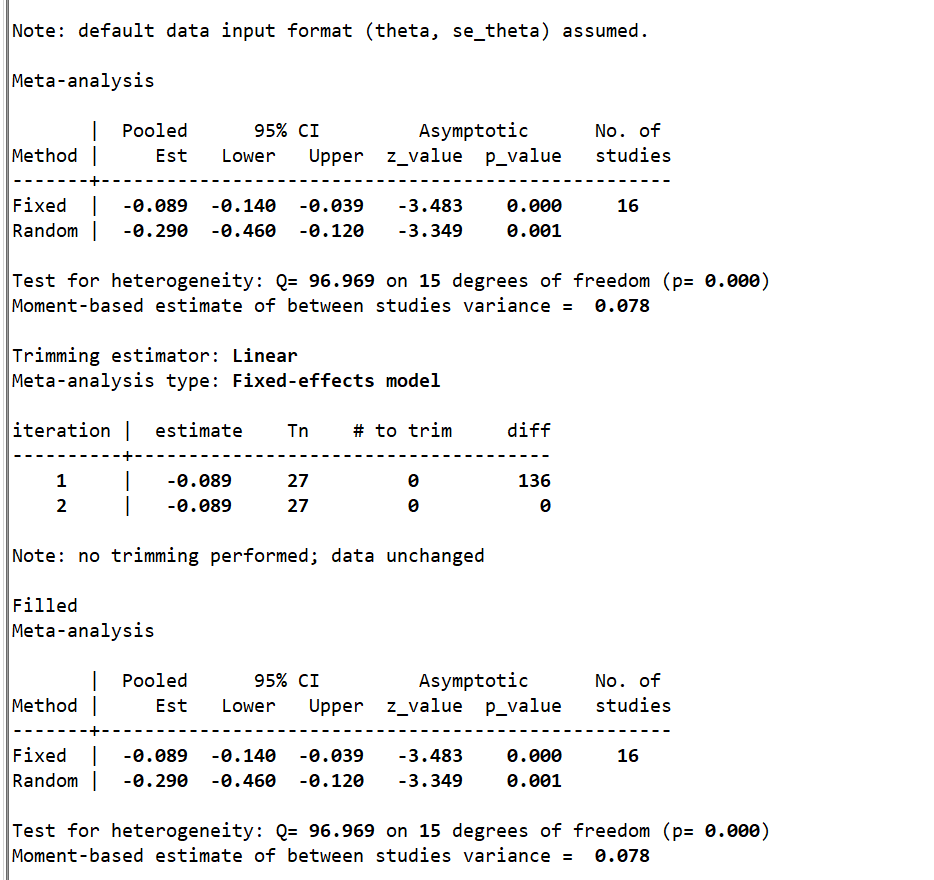

Supplement: S2 File — (ZIP) [file pone.0315504.s003.zip › S2 File/S3 Fig/Trim-and-Fill Data for FBG.jpg]
